# Relationships between Structures of Condensed Tannins from Texas Legumes and Methane Production During In Vitro Rumen Digestion

**DOI:** 10.3390/molecules23092123

**Published:** 2018-08-23

**Authors:** Harley Naumann, Rebecka Sepela, Aira Rezaire, Sonia E. Masih, Wayne E. Zeller, Laurie A. Reinhardt, Jamison T. Robe, Michael L. Sullivan, Ann E. Hagerman

**Affiliations:** 1 Division of Plant Sciences, University of Missouri, 110 Waters, Columbia, MO 65211, USA; naumannhd@missouri.edu; 2 Department of Chemistry & Biochemistry, Miami University, Oxford, OH 45056, USA; rjsepela@ucdavis.edu (R.S.); aira.rezaire@gmail.com (A.R.); masihse@miamioh.edu (S.E.M.); 3 USDA-ARS, U.S. Dairy Forage Research Center, Madison, WI 53706, USA; wayne.zeller@ars.usda.gov (W.E.Z.); laurie.reinhardt@ars.usda.gov (L.A.R.); jamison.robe@ars.usda.gov (J.T.R.); michael.sullivan@ars.usda.gov (M.L.S.)

**Keywords:** proanthocyanidins, condensed tannins, thiolysis, NMR spectroscopy, ultrahigh-resolution negative mode MALDI-TOF mass spectrometry, antioxidant, ORAC assay, *Acacia*, forage legume

## Abstract

Previous studies showed that a series of purified condensed tannins (CTs) from warm-season perennial legumes exhibited high variability in their modulation of methane production during in vitro rumen digestion. The molecular weight differences between these CTs did not provide correlation with either the in vitro CH_4_ production or the ability to precipitate bovine serum albumin. In an effort to delineate other structure-activity relationships from these methane abatement experiments, the structures of purified CTs from these legumes were assessed with a combination of methanolysis, quantitative thiolysis, ^1^H-^13^C HSQC NMR spectroscopy and ultrahigh-resolution MALDI-TOF MS. The composition of these CTs is very diverse: procyanidin/prodelphinidin (PC/PD) ratios ranged from 98/2 to 2/98; cis/trans ratios ranged from 98/2 to 34/66; mean degrees of polymerization ranged from 6 to 39; and % galloylation ranged from 0 to 75%. No strong correlation was observed between methane production and the protein precipitation capabilities of the CT towards three different proteins (BSA, lysozyme, and alfalfa leaf protein) at ruminal pH. However, a strong non-linear correlation was observed for the inhibition of methane production versus the antioxidant activity in plant sample containing typical PC- and PD-type CTs. The modulation of methane production could not be correlated to the CT structure (PC/PD or cis/trans ratios and extent of galloylation). The most active plant in methane abatement was *Acacia angustissima*, which contained CT, presenting an unusual challenge as it was resistant to standard thiolytic degradation conditions and exhibited an atypical set of cross-peak signals in the 2D NMR. The MALDI analysis supported a 5-deoxy flavan-3-ol-based structure for the CT from this plant.

## 1. Introduction

**Figure 1:**
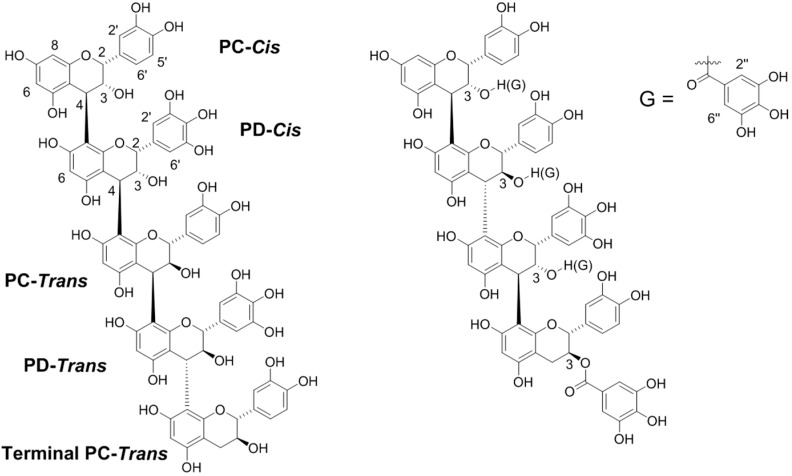
Common representation of condensed tannin structures. Left structure: PC = procyanidin; catechin (*trans* isomer) or epicatechin (*cis* isomer). PD = Prodelphinidin; gallocatechin (*trans* isomer) or epigallocatechin (*cis* isomer). In addition, hydroxyl groups, particularly on the C-3 hydroxyl, may be esterified with a galloyl (G) group (structure on the right). Carbons 2, 3, and 4 of the C ring, 2′ and 6′ of the B ring, 6 and 8 of the A ring, and 2” and 6” of the D ring are labeled.

Condensed tannins (CTs, proanthocyanidins) consist of oligomers and polymers of flavan-3-ol subunits. Variations in hydroxylation patterns, *cis*- and *trans*-configuration of C-ring substituents, interflavan bond connections, mean degree of polymerization (mDP), and extent of esterification have been described for natural condensed tannins from various plants [1] (Figure 1).

Condensed tannins in human foods and beverages, including berries, cocoa, and wine, have been described in detail [2] but less is known about the CTs found in natural forages. Early studies on tannin-rich forages focused on diminished nitrogen utilization associated with protein binding by tannins during digestion [3] but more recent studies have revealed beneficial effects of tannins on domestic ruminants. For example, consumption of CT-containing feedstuffs can protect forage/microbial-generated protein during digestion and result in higher nitrogen utilization [4], improved meat and milk quality [5] and increased wool production [6]. Additional biological effects associated with CT-rich feeds include the mitigation of intestinal nematode proliferation [7], and the reduction in the incident of bloat [8] and abatement of methane production during digestion [9]. Ruminant animals contribute significantly to the production of methane during rumination and this greenhouse gas contributes to the increase of global temperatures [10]. Production of methane translates into lost energy which can negatively affect ruminant productivity [11], so identification of feeds that reduce methane production is economically favorable as well as environmentally beneficial.

Tannin-rich plants diminish ruminal methane production both in vitro [12,13] and in vivo [14,15,16]. Adding crude tannin to tannin-free forage also reduces methane production [17,18]. Notably, meta-analysis of the effects of tannin on methane production identified a significant relationship between level of tannin and methane production [9]. This analysis suggested that there is a broad-based tannin effect on methane production since studies that used both hydrolyzable and condensed tannins were included in the dataset. The authors noted that supplementing a diet with exogenous tannin may not be as effective as supplying a tannin-containing feed, presumably because the tannin is released at different rates and in different forms from natural vs. amended feeds. It is important to develop models that predict the relationship between tannin content of feeds and methane production because high levels of tannin in the diet can be detrimental to the animals [19]. Furthermore, the costs of detoxifying tannin and other natural products can outweigh the economic benefits of reducing methanogenesis [20]. Attempts to develop models have had limited success to date due to lack of data linking chemical details to bioactivity [21,22].

Although both hydrolyzable and condensed tannins appear to have a role in limiting methane production during rumination, the current study is focused only on CTs because of their widespread distribution among forages suitable for domestic ruminants [8]. Most of the studies to date provide limited dose-response information, and almost no information on tannin structure or chemical properties. In one quantitative in vitro study that examined a variety of CT-rich species, Naumann et al. showed that total condensed tannins vs. methane production gave a correlation of R^2^ = 0.44, suggesting that only 44% of the reduction in methane production is explained by total CT [23]. Other authors have proposed tentative relationships between methane production and CT structural features such as the degree of hydroxylation of the B ring, the *cis/trans* isomer ratio, or the molecular weight of the CT [24,25,26,27], but a strong predictive model has not been developed.

Tannins have several potent bioactivities that could be responsible for their role in ruminant nutrition. In addition to their well-known role as protein precipitating/binding agents [28], tannins are antioxidants [29]. Relatively few studies have attempted to correlate tannin bioactivity with suppression of methane production, although weak relationships between protein binding activity and methanogenesis have been reported in a few systems [30,31]. Studies correlating antioxidant activity of polyphenols to methane production have not been published to date.

In this study, we explore the role of CT structure on methane production in a model rumen system using a collection of CT-rich dryland legume species (Table 1) that have a range of abilities to diminish methane production in an in vitro model rumen [23]. We used several methods to evaluate the tannin structure, including degradation to anthocyanidins, quantitative thiolysis, ultrahigh-resolution negative mode MALDI-TOF MS, and 2D NMR spectroscopy of purified CTs from these legumes. We used the oxygen radical absorbance capacity test (ORAC) method to establish the antioxidant activity of each isolated CT [32] and correlated that activity to the methane production data from earlier studies [23]. We extended protein precipitation studies with CTs in this series of forage legumes to include not only bovine serum albumin, but also lysozyme and alfalfa leaf protein with assays conducted at ruminal pH.

## 2. Results

### 2.1. Degradative and NMR Analysis of Purified CTs

Table 1 contains data from the acidic methanolysis to yield anthocyanidins, a classic method for estimating the composition of condensed tannins [33]. The λ_max_ of the mixture of reaction products provides general information about the predominance of subunits yielding delphinidin (λ_max_ 548 nm), cyanidin (λ_max_ 538 nm) or other anthocyanidins. For typical CT, acid methanolysis breaks the interflavan bond and any ester bonds, so that cyanidin is produced from catechin, epicatechin, and (epi)catechin gallate extender units while delphinidin is produced from (epi)gallocatechin and (epi)gallocatechin gallate [34]. The CT from these plants is categorized as prodelphinidin (PD) (*Lespedeza cuneata, Mimosa*, *Desmanthus*, *Neptunia*), procyanidin (PC) (*Leucaena*), or mixed (λ_max_ intermediate between 548 nm and 538 nm) (*Desmodium*, *Lespedeza stuevei*) (Table 1). The CT from *Acacia* was neither PC- nor PD-based, but produced small amounts of an anthocyanidin with λ_max_ typical of guibourtinidin, a 5-deoxy anthocyanidin (Table 1).

Thiolysis is a degradative method of analysis that provides more structural detail than acid methanolysis (Table 2 and Table S1). The data provide quantitative procyanidin:prodelphinidin (PC/PD) ratios [25] that complement and extend the methanolysis results. The CTs identified as PD by methanolysis contain less than 15% of the cyanidin-type subunits, while the PC from *Leucaena* contains < 2% delphinidin-type units (Table 2). The mixed CTs (*Desmodium*, *Lespedeza stuevei*) contain similar amounts of both subunits (Table 2).

The thiol products retain the original stereochemistry at C2 and C3, so *cis*/*trans* ratios for the CT can be calculated from thiolysis data. For these plants, *cis* stereochemistry (epicatechin pattern) is dominant (Table 2). Furthermore, thiolysis does not cleave the gallate ester bond so % galloylation can be determined from thiolysis data [34]. A predominance of the CT subunits are galloylated in the CT from *Desmanthus* while there are essentially no galloyl esters in *Desmodium* or the two *Lespedeza* species (Table 2).

Previous studies have shown that significant CT structural information (PC/PD and *cis*/*trans* ratios) can be obtained through integration of corresponding cross-peaks (Tables S4 and S5) in the ^1^H-^13^C HSQC NMR spectra of purified CT samples and highly corroborate results obtained via thiolytic degradation [35]. Analysis of the ^1^H-^13^C HSQC NMR spectra for these CTs showed that all the samples were of high purity indicated by the absence of significant non-CT component cross-peaks (Figures S1–S7). Detailed analysis of the information derived from ^1^H-^13^C HSQC NMR spectra is provided in the supplemental section (Tables S4 and S5, Figures S1–S9).

For the most part, the structural information obtained from thiolysis and NMR was very consistent. For PC:PD ratios, thiolytic and ^1^H-^13^C HSQC NMR analyses were no more than 4% different (Table 2). The *cis/trans* ratios were consistent within 10% (Table 2), and the % galloylation was consistent within 15%.

The mean degree of polymerization (mDP) for CT can be estimated through integration of the cross-peak volumes in NMR or from the peak area ratios for the extender and terminal units from thiolysis (Table 2). The two methods proved to be very consistent for all of the plants, except *Leucaena* (Table 2). In general, the CT from the plants in this study had mDP ranging from about 6 (*Desmanthus*) to about 20 (*Desmodium*). *Leucaena* was an exception, with a very high mDP (39) estimated by thiolysis, although NMR suggested a more typical mDP of about 6. The thiolysis data can overestimate mDP if the terminal units are heterogeneous leading to underestimation of some minor terminal units, but close inspection of the chromatograms from *Leucaena* did not provide any indication of minor terminal units. If the tannin preparation is contaminated with monomeric flavan-3-ols, the thioloysis method can underestimate mDP, but we eliminate this potential problem by running control samples of unthiolyzed tannin that reveal the presence of free flavan-3-ols. Similar to thiolysis, the NMR method does not always provide a useful measure of mDP. In some cases, the cross-peak signals of the terminal methylene C-H bonds suffer from low signal-to-noise ratios, increasing inherent error in mDP determination as mDP values increase. In these cases, no estimate of mDP was made based on 2D NMR data (*Desmodium*, Figure S1). In other cases, the signal arising from the terminal methylene C-H bonds is disrupted by the neighboring solvent (DMSO) peak, as exemplified by the spectrum for *Lespedeza cuneata* (Figure S3). In this case, we integrated the unperturbed, more downfield cross-peaks, and adjusted the formula for the mDP calculation to account for this modification. 

The tannin from *Acacia* could not be analyzed by thiolysis. The reaction product chromatograms were dominated by a large peak characteristic of undegraded tannin, indicating that the reaction conditions are not sufficiently vigorous to degrade this CT. We tentatively concluded that the *Acacia* CT mainly comprises 5-deoxy flavan-3-ols (robinetinidol, fisetinidol, and/or guibourtinidol), based on the acid methanolysis data and the resistance of the *Acacia* CT to degradation [36,37,38]. This conclusion is supported by the 2D NMR spectral data. First, the H/C-6/8 set of cross-peaks arising from the A-ring of PC and PD subunits has a diminished presence relative to other NMR cross-peaks indicating that the phloroglucinol substitution pattern of the A-ring is not present to an appreciable extent. Second, cross-peaks typically assigned to a *para*-substitued phenolic B-ring, appear at 6.4/128 ppm in the NMR spectra of the *Acacia* CTs, indicating the guibourtinidol or afzelechin subunit substitution pattern. Lastly, the H/C-4 cross-peaks arising from extender subunits of the CT appear at chemical shifts that do not coincide with those of CTs composed of PC/PD subunits. In addition, the 2D NMR spectra exhibited a series of unusual and previously undocumented cross-peak signals, with the most unusual peaks found in the region where aliphatic signals, such as alpha carbonyl or allylic C-H, are usually observed (Figures S8 and S9). This may be due to an alkyl modification of the general CT structure. These cross-peaks are present even after multiple isolation and sequential purification attempts, so we believe that they do not arise from impurities co-eluting during the purification steps. 

### 2.2. MALDI-TOF Mass Spectrometric Analysis

**Figure 2:**
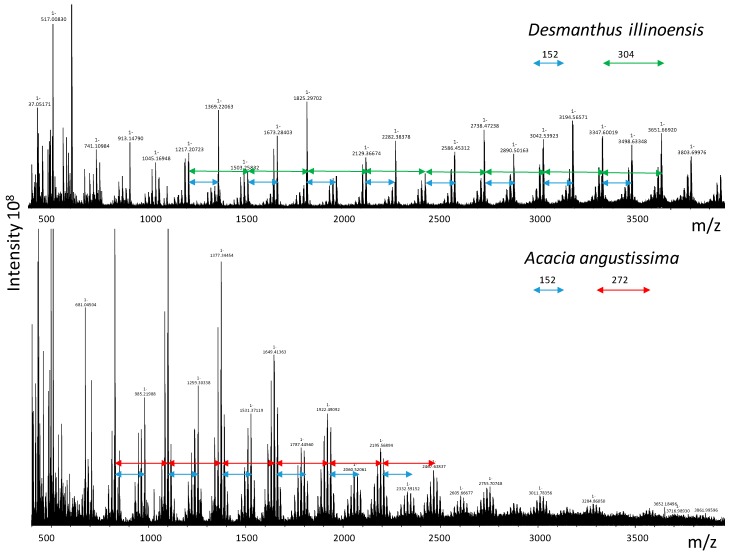
Representative ultrahigh resolution negative mode MALDI-MS data for CT. The top spectrum was obtained with CT from *Desmanthus illinoensis* and the bottom spectrum with CT from *Acacia angustissima*. The mass intervals between the clusters of peaks represent the characteristic subunits for the CT, with an interval of 304 typical of (epi)gallocatechin, an interval of 272 typical of fisetinidol, and an interval of 152 typical of gallate ester modification.

MALDI-TOF MS has been widely used to assess the composition of high molecular weight CTs with complex structural subunits [39,40]. In most previous studies, the data were collected in the positive ion mode using lower resolution instruments, and interpretation relied on finding intervals in the spectra characteristic of different types of subunits. An interval of 288 indicates (epi)catechin subunits (PC) while an interval of 304 is characteristic of (epi)gallocatechin subunits (PD) [40]. For the plants in this study, the intervals in the spectra (Figure 2, Table 3) confirm the CT compositions obtained by thiolysis and NMR. For example, the spectrum of *Desmanthus* is dominated by intervals of 304 and 152 mass units, consistent with the highly galloylated PD-type tannin identified by thiolysis and NMR. In addition to the major intervals, clusters of peaks separated by intervals of 16 mass units are clearly seen in each spectrum, consistent with different degrees of hydroxylation (catechin vs. gallocatechin, etc.). Although PC and PD subunits are easily distinguished by mass spectrometry, stereochemical differences are not detected so MALDI cannot be used to calculate the *cis*/*trans* ratios or other stereochemical features of CT.

Interpretation of positive ion mode MS data can be complicated by the ambiguity of protonated vs. metallated (Na^+^, K^+^) forms of the analyte [41,42]. In this study, we used Fourier transform ion cyclotron resonance (FT-ICR) ultrahigh-resolution negative ion MALDI-TOF MS analysis, which not only eliminates the ambiguity of the cation species but also provides absolute identification of subunit composition by exact mass (Table 4). For example, *Desmanthus* CT comprises molecules with up to one gallate per flavan-3-ol subunit while *Neptunia* CT had only 0.2 gallate per favan-3-ol (Table 4). Unlike thiolysis or NMR, MALDI does not provide an accurate DP for the polymers, because ionization of higher molecular weight species is less efficient than that of smaller molecules. For the CT in this study, signals in the 3000 (DP ~10) to 4000 (DP ~14) were easily detected for all the plants except *Acacia*. For example, the predicted mean molecular weight of *Desmanthus* CT (mDP 6) is about 2500, consistent with the pattern of strong MALDI signals between 500 and 4000 mass units (Figure 2). The MALDI data for *Leucaena* has a peak distribution from about 500 to 4000 mass units similar to that of *Desmanthus*, suggesting the NMR estimate of mDP 6 is more accurate than the thiolysis estimate of mDP 39 for this CT (Table 2).

The ultrahigh-resolution MALDI-TOF data were particularly useful for the *Acacia* CT that was resistant to analysis by NMR or thiolysis. Intervals of 272 and 152 dominated the spectrum for this CT, consistent with a structure based on the 5-deoxy flavan-3-ol fisetinidol with a moderate degree of galloylation (Figure 2, Table 3). Using exact masses we identified CT fragments comprised of fisetinidol and small amounts of guibourtinidol with gallate esters (Table 4). Thus, the MALDI analysis is consistent with the hypothesis that the unique CT of *Acacia* is a 5-deoxy CT.

### 2.3. Antioxidant Activity

**Figure 3:**
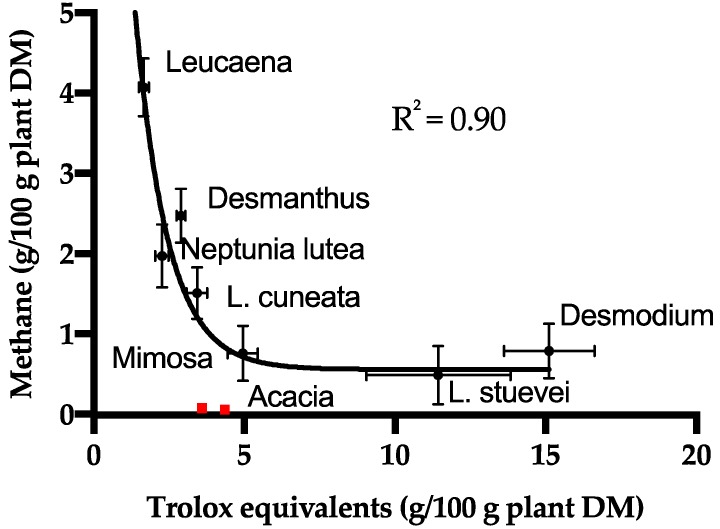
Relationship between methane production [23] and antioxidant activity for the forages. The non-linear exponential fit (Y = 11.64(exp(−0.772X)) + 0.6433) does not include the *Acacia* CT data (red markers). The points are average for the ORAC method (*n* = 3) and the in vitro gas production (*n* = 3) and the error bars indicate standard deviations.

The ORAC method estimates the hydrogen atom transfer (HAT) potential of the putative antioxidant [43] by comparing the activity of the material to the well-known standard antioxidant Trolox [32]. For this set of plants, Trolox equivalents (TE) per g of CT ranged from 0.3 TE/g (*Neptunia*) to 1.5 TE/g (*Desmodium*) (Table S2). We excluded the poorly characterized *Acacia* CT from the analysis and attempted to correlate the molecular features of the CT (PC:PD ratio, % trans, % galloyl, mDP) to the antioxidant activity. Although previous studies found that galloylation increased the antioxidant activity of flavan-3-ols [44], we did not find strong relationships between any structural feature and antioxidant activity for these CT (Supplemental Files). Although there were no correlations between CT structure and antioxidant activity, there was a strong nonlinear correlation (R^2^ = 0.90) between antioxidant activity (TE per g of plant tissue) and methane production (g CH_4_ per kg of plant tissue) when the *Acacia* CT was excluded (Figure 3). The *Acacia* is exceptional because it is a very effective inhibitor of methane production despite its moderate antioxidant activity (Figure 3).

### 2.4. Protein Precipitation Activity

In previous studies, we examined the protein precipitation capabilities of the purified CTs from Texas legumes using bovine serum albumin (BSA) in acetate buffer at pH 4.9 [45], and found no relationship between precipitation, CT molecular weight, and methane abatement. To determine whether the use of the model protein BSA biased this conclusion, we performed additional protein precipitation assays using model proteins, lysozyme, and alfalfa leaf protein at pH 6.5 (rumen pH) [46] (Figure S10a–f, Table S3). We did not find a clear correlation of the protein precipitation capacity and the level of methane inhibition (Figure S11a–f). Thus, we conclude that the ability of the CT to precipitate forage protein is not a major driving force relative to the inhibition of methane production.

In our analysis we compared methane production during in vitro digestion of intact tissue to chemical characteristics of extracted, soluble CT. Plant tissue may contain insoluble and cell wall-bound CT and other chemical (e.g., alkaloids) and physiochemical (e.g., lignified cell walls) attributes unrelated to CT content. Our correlation analysis can provide insights into possible mechanisms of methane abatement but does not provide proof of activity for the components tested. Earlier workers have noted that natural feeds more effectively inhibit methane production than tannin-amended feeds [9], suggesting that features of the intact tissue help to inhibit excess methanogenesis.

## 3. Discussion

### 3.1. Methods to Determine CT Structure

The three high-resolution methods were used to establish CT structure. Data obtained from the three methods were complementary and yielded very consistent results. Each method has strengths and weaknesses. Thiolysis is the most accessible of the methods, since HPLC with DAD is highly available and the chemical degradation method is simple. Thiolysis is very sensitive, requiring only a few milligrams of sample. It can be carried out on purified CT, unpurified CT extracts, or directly on plant tissue [47], making it particularly useful for screening plant collections. Unfortunately, a commercial library of standards is not available, making it necessary for individual laboratories to use methods such as HPLC-MS and published CT compositions to develop an internal library. Thiolytic analysis has traditionally been limited to B-type CT with 4- > 8 or 4- > 6 interflavan bonds [48], but has recently been modified to analyze A-type crosslinked CT [49]. Thiolysis is not useful for the chemically-resistant 5-deoxy CT [37], such as the *Acacia* CT in this study.

NMR spectroscopy is particularly useful because it is nondestructive and not dependent on derivatization. Samples can be recovered after analysis, and the method does not suffer from potential differences in the rates of derivatization among substrates or the production of side-products under derivatization conditions. CT samples must be reasonably pure (>70%) to avoid inclusion of overlapping cross-peaks from non-CT components interfering with integration values. The relative purity of sample can be rapidly assessed by the observations of presence or absence of non-CT cross-peaks, providing a definite advantage over both thiolysis and MALDI-TOF analyses. In cases where the measurement of a minor component present is desired, small signal-to-noise ratios of the cross-peaks of minor components undergoing analysis can lead to increasing the inherent error in the measurement. Both ^13^C and ^1^H-^13^C HSQC NMR experiments require several hours or overnight acquisitions which limits access on multi-user instruments. NMR techniques are not as sensitive as thiolysis and mass spectrometry and require more sample for analysis on common instruments. 

MALDI-TOF analysis is generally available only through a mass spectrometry facility, making it less accessible than thiolytic degradation. Positive-mode MALDI-TOF MS has been used in several other studies of CT structure to confirm data from other analytical methods [40,42]. Here, we used ultrahigh-resolution (15T Bruker SolariXR FT-ICR, Bruker Corp., Billerica, MA, USA) negative-mode MALDI MS and were able to confirm details of CT chemical formulae that were previously inaccessible. The ultrahigh resolution of the MALDI analysis allowed us to assign exact structures to the peaks noted in the spectra. For example, we calculated the stoichiometric ratio for ester groups on the galloylated CTs from the exact structures established by MALDI analysis. The 1:1 galloyl:flavan-3-ol ratio typical of species in the highly-modified *Desmanthus* CT is much larger than the ratios (0.2:1, 0.4:1) obtained for the less highly esterified CT from *Neptunia* or *Leucaena*. For each species, the galloyl:flavan-3-ol ratio from the exact structure is consistent with the % galloyl determined by thiolysis or NMR (Table 2 and Table 4). Similarly, the PC/PD ratios determined by thiolysis or NMR reflect the exact structures determined by MALDI, as illustrated by the cat_4_-gallocat_5_ exact structure identified in *Desmodium* CT, which has a PC:PD ratio of about 55:45 (Table 2 and Table 4).

Most importantly, MALDI analysis of the *Acacia* CT allowed us to establish structural information that was not available from thiolysis or NMR. The *Acacia* tannin is structurally diverse, with three types of subunits and a stoichiometric ratio of one galloyl ester per four flavan-3-ols corresponding to 25% galloylation (Table 4). As suggested by its recalcitrance to thiolysis, most of the subunits in the *Acacia* CT are 5-deoxy flavan-3-ols, but the individual structures identified from the exact mass data suggested that catechin was always the terminal unit of the 5-deoxy CTs.

Based on this direct comparison of methods to determine structures of CT, we conclude that thiolysis and NMR provide very similar information but that MALDI FT-ICR MS provides unique structural information about these complex natural products. While thiolysis and NMR yield quantitative composition and unambiguous stereochemical assignments based on bulk CT properties, ultrahigh resolution MALDI can be used to assign structures of specific molecular species. It is still not possible to purify CT to single molecular species, but, as demonstrated here, the pure polydisperse mixtures of CT purified by Sephadex LH-20 chromatography [50] can be described in detail using a combination of chemical and spectroscopic tools. Thiolysis and NMR provide information about average degree of polymerization of CT, but methods for obtaining more accurate information about molecular size and heterodispersity of CT are needed to further advance our knowledge of CT structure.

### 3.2. Chemotaxonomy

The legumes (family Fabaceae) analyzed here represent three taxonomic groupings [51] (Table 1). Similarities in secondary chemistry are often found within taxonomic groups, reflecting genetic similarities in enzymology and metabolic regulation [52]. Chemotaxonomic trends have previously been noted for polyphenols including flavonoids and tannins [47,53,54]. In this study, the CT from plants in the subfamily Papilionoideae are not galloylated, while CT from the Mimosoidae contain > 20% galloylation (Table 2 and Table 4). Plants from the Papilionoideae are more likely to have mixed PC/PD, while Mimosoidae are more likely to have PD-type CT. Interestingly, *Lespedeza cuneata*, which has PD-type CT, is quite distinct from the closely related species *Lespedeza stuevei* or *Desmodium*, which contain mixed PC/PD CT (Table 1 and Table 2).

*Acacia* is distinguished taxonomically from the other Mimosoidae (Table 1) and is also chemically distinct. *Acacia* CT is unique compared to the other plants in our set because of it is predominantly comprised of 5-deoxy flavan-3-ol subunits (Table 3 and Table 4, Figure 2). The widespread occurrence of 5-deoxy flavonoid derivatives in *Acacia* was reported in many early surveys of the genus [51], and more recent studies have reported 5-deoxy CTs in *A. mangium* and *A. mearnsii* bark extracts [55,56]. One conflicting report found that CTs from leaves of *A. karroo* and *A. grandicornuta* were simple prodelphinidins or procyanidins, respectively, with no 5-deoxy-type subunits [57]. Our new structural analysis provides key information for the polyphenols from this important group of plants. In particular, the MALDI analysis shows that the CT is predominantly profistetinidin-based, with 25% galloylation. The *Acacia* CT does contain catechin units, which may serve as the terminal units of the 5-deoxy flavan-3-ol polymers. New methods of NMR and HSQC analysis that utilize phosphate-labeled CT offer promise for providing additional details about the structure of *Acacia angustissima* foliage CT [58]. 

### 3.3. Methane Abatement

Many studies have demonstrated that forage plants with diverse natural products decrease gas production by ruminants, including methane emissions [59]. Previous studies have identified *Mimosa* spp. and *Acacia mearnsii* as tannin-rich legumes that decrease methane production, but mechanisms of action have not been established [15,19,60]. Biological activities of tannins are often attributed to their characteristic ability to bind and precipitate protein, but our data suggest that the ability of the CT to precipitate forage protein is not directly related to the inhibition of methane production. For the plants studied here there is a strong nonlinear correlation between radical quenching activity and methane production, suggesting that CT, or other antioxidants contained in the forages may affect rumen microbial populations and their ability to carry out redox chemistry required for methane production.

The CT from the plants in this study that were most effective at reducing methane production in vitro (*Desmodium, Lespedeza stuevei, Mimosa*, *Acacia*) do not have any single common functional or structural feature. Condensed tannins from two of the plants that reduced methane production, *Desmodium* and *Lespedeza stuevei,* have similar compositions. Both CT have mixed PC and PD composition and trace levels of galloylation, features not previously identified as important in the context of methanogenesis. Earlier proposals that higher molecular weight CTs more effectively inhibit methane production were based on work with CT with apparent chain lengths ranging from about 15 to 70 [25]. It is difficult to compare that work to our CT with chain lengths from 6 to 20, although *Desmodium* CT (mDP 20) had high methane abatement activity. *Leucaena* had a very high mDP based on thiolysis data, but a much lower mDP based on NMR and MALDI analysis, and had little effect on methane production. 

*Mimosa* CT is predominantly PD, has a moderate chain length, and an intermediate level of galloylation. Several earlier studies have noted a relationship between PD-type CT and diminished methane production [24,26]. The trihydroxy substitution pattern of PD-type tannins may confer particular reactivity that disrupts methanogenesis.

In our work, *Acacia* stands out for its high potential to abate methane production (Table 1) [23]. Based on the structural data obtained so far, we propose that the activity of *Acacia* CT in the rumen is at least in part a consequence of its chemical structure and characteristics. The interflavan bond in a typical CT is susceptible to attack because the 5-hydroxyl group adjacent to the bond is able to electronically contribute to the stabilization of the transient cation/quinone methide intermediate arising from acid catalyzed interflavan bond cleavage. CT comprised of 5-deoxy flavan-3-ol subunits lack this hydroxyl group and are less chemically susceptible to acid-catalyzed interflavan bond cleavage than typical CT [37]. We suggest that, relative to PC/PD CTs, *Acacia angustissima* tannins may have an unusually long lifetime in the rumen because of their chemical stability. A priority for future research is a more detailed determination of the structure of the *Acacia* CT and tests of its chemical stability under rumen conditions. Persistent, long-lived CT in the rumen may serve as a selective toxin that eliminates methanogens [61,62]. In addition to direct toxicity, the metal binding activity of tannins [7,63] could alter the availability of essential or toxic metals to rumen microbes especially if the CT has a long lifetime in the rumen. We propose that tannins modulate the rumen microbiome and as a consequence control methane production, and that *Acacia* tannins are especially active in the rumen because of their chemical stability and prolonged life times. Further studies using “omics” tools to explore the rumen microbiome under the influence of chemically well-defined tannins will be required to test this hypothesis.

## 4. Materials and Methods

### 4.1. Chemicals

The 2,5-dihydroxybenzoic acid was purchased from Hewlett Packard (Palo Alto, CA, USA, MALDI Quality Matrix Solutions). Sephadex LH-20, bovine serum albumin and chicken egg white lysozyme were from Sigma (St. Louis, MO, USA) and anthocyanidin chlorides from Chromadex (Irvine, CA, USA). Fluorescein (FLNA) was obtained from Spectrum (Stamford, CT, USA), Trolox (97%) from Acros (Bridgewater, NJ, USA), and 2,2′azobis(2-amidinopropane) HCl (AAPH) from Polysciences Inc., (Warrington, PA, USA). Solvents were HPLC grade and all other chemicals were reagent grade.

### 4.2. Tannin Purification

The harvesting and preparation of these North American native warm-season perennial legume samples (Table 1) have been previously described [23]. The plants included representatives of subfamily Papilionoideae (*Desmodium paniculatum* (panicledleaf ticktrefoil), *Lespedeza stuevei* (tall lespedeza), and *Lespedeza cuneata* (sericea lespedeza)), subfamiliy Mimosoideae subtribe Mimoseae [*Leucaena retusa* (littleleaf leadtree), *Desmanthus illinoensis* (Illinois bundleflower), *Mimosa strigillosa* (powderpuff), *Neptunia lutea* (yellowpuff)], and subfamily Mimosoideae subtribe Acacieae (*Acacia angustissima var. hirta* (prairie acacia)). Two ecotypes of *Acacia* were analyzed, one originating in the South Texas Plains (AA-STX) and the other from the Cross Timbers ecosystem (AA-STP5). All leaves were dried at 55 °C in a forced air oven for 48 h before grinding the samples to pass a 1 mm screen in a sheer mill and storing at −40 °C.

Tannin was prepared by standard methods [50]. Lipids were removed from the leaves with diethyl ether before extracting total polyphenols with 70% (*v*/*v*) acetone:water. Acetone was removed by rotary evaporation and the material was applied to a Sephadex LH-20 column equilibrated with methanol. After eluting small phenolics and nonphenolics with methanol, the column was washed with 70% acetone to recover the condensed tannins. The acetone was removed from the eluate by rotary evaporation and the tannin was freeze dried and stored at −40 °C [45]. The extinction coefficient (E_280_^1 mg/mL^) was determined for each purified tannin using serial dilutions of 1.500 mg/mL aqueous solutions.

### 4.3. Anthocyanidins

The anthocyanidins produced by heating in alcoholic methanol were determined for each tannin by dissolving about 1 mg of the tannin in 200 µL of 6.25% HCl:methanol (*v*/*v*) and incubating the solution on a heating block at 70 °C for 30 min. The products were diluted with 200 µL methanol and analyzed directly by UV–VIS spectrometry (Agilent 8453, Santa Clara, CA, USA) to obtain the λ_max_ of the mixture of products. The spectral features of samples were compared to commercial standards of the six most common anthocyanidins (delphinidin, cyanidin, pelargonidin, robetinidin, fisetinidin, and guibourtinidin) [64].

### 4.4. Thiolysis

Purified CTs were analyzed by thiolysis according to published procedures [39,47]. Briefly, approximately 1 mg of tannin was dissolved in 200 µL of methanol containing 30 µL of the HCl reagent (32% (*v*/*v*) HCl in methanol) and 72 μL of the thiol reagent (5% (*v*/*v*) toluene-α-thiol in methanol) and incubated at 40 °C for 30 min. The thiolytic degradation products were analyzed by HPLC using an Agilent 1100 HPLC with ChemStation Rev. A.09.03 software (Agilent, Santa Clara, CA, USA). The column was an Agilent Zorbax PR-8 column (Agilent, Santa Clara, CA, USA), 4.6 mm × 150 mm with 5 μM packing. The gradient program employed 0.13% (*v*/*v*) trifluoroacetic acid in water and 0.10% (*v*/*v*) TFA in acetonitrile [47]. Reaction products were detected at 220 nm and were identified by their retention times and spectral characteristics compared to authentic standards [39,47]. Products were quantitated based on peak areas and converted to moles of extender and moles of terminal units. The chromatograms from control samples that did not contain the reagents were used to confirm that all of the tannin was degraded by thiolysis, and to confirm that the tannin did not contain any flavan-3-ol contamination that would interfere with terminal unit determination.

### 4.5. NMR Spectroscopy

^1^H, ^13^C, and ^1^H-^13^C HSQC NMR spectra were recorded at 27 °C on a BrukerBiospin DMX-500 (^1^H 500.13 MHz, ^13^C 125.76 MHz, Bruker Corp., Billerica, MA, USA) instrument equipped with TopSpin 3.5 software (Bruker Corp., Billerica, MA, USA) and a cryogenically cooled 5-mm TXI ^1^H/^13^C/^15^N gradient probe in inverse geometry. Spectra were recorded in DMSO-*d*_6_ and were referenced to the residual signals of DMSO-*d*_6_ (2.49 ppm for ^1^H and 39.5 ppm for ^13^C spectra). ^13^C-NMR spectra were obtained using 1K scans (acquisition time 56 min). For ^1^H-^13^C HSQC experiments, spectra were obtained using between 200 and 620 scans (depending on sample size and instrument availability) obtained using the standard Bruker pulse program (hsqcetgpsisp.2) with the following parameters: Acquisition: TD 1024 (F2), 256 (F1); SW 16.0 ppm (F2), 165 ppm (F1); O1 2350.61 Hz; O2 9431.83 Hz; D1 = 1.50 s; CNST2 = 145. Acquisition time: F2 channel, 64 ms, F1 channel 6.17 ms. Processing: SI = 1024 (F2, F1), WDW = QSINE, LB = 1.00 Hz (F2), 0.30 Hz (F1); PH_mod = pk; Baseline correction ABSG = 5 (F2, F1), BCFW = 1.00 ppm, BC_mod = quad (F2), no (F1); Linear prediction = no (F2), LPfr (F1). Sample sizes used for these spectra ranged from 3–10 mg providing NMR sample solutions with concentrations of 6–20 mg/mL.

### 4.6. MALDI-TOF Mass Spectrometry

Solutions of purified CT samples were prepared (15 mg/mL) using reagent grade methanol. The CT sample (1 μL) was mixed with 10 μL of DHB matrix solution (0.1 M in acetonitrile:water, 1:1, containing 0.1% formic acid). A 1 μL aliquot of this sample-analyte mixture was deposited on the plate and allowed to dry before inserting in the Bruker 15T FR-ICR MALDI-MS. Calibration was run on a standard peptide mix (Bruker Daltonics, Billerica, MA, USA) in the negative ion mode. Typically, 20% of the Yag/Nd (351 nm) laser power was used for the spectral acquisition.

### 4.7. Antioxidant Activity

The ORAC assay was used to evaluate antioxidant potential [32]. Fluorescein (30 nM) was prepared in 75 mM phosphate pH 7 (PBS) and stored in the dark at 4 °C. The Trolox stock solution (5 mM in reagent grade methanol) was stored in the dark at 4 °C and diluted ten-fold in PBS for use. A 60 mM solution of the radical initiator (AAPH) was prepared immediately before use. Tannin samples were dissolved in water and diluted to an appropriate working concentration (0.1–10 μg/mL based on the absorbance at 280 nm and the extinction coefficients described above) after preliminary ORAC analyses to establish the correct concentration range for each tannin. The reactions were performed in triplicate in 96 well plates, with each 400 µL sample containing 25 nM fluorescein, an appropriate concentration of the antioxidant, and 5 mM AAPH. Immediately after adding the AAPH to the samples the plate was inserted into the plate reader (Biotek, Winooski, VT, USA) and the kinetics of fluorescence decay were recorded with excitation at 685 nm and emission at 520 nm. A standard curve with 0–8.3 μM Trolox was run with every set of samples. At the end of the 1.5 h reaction, the area under the curve was calculated for each reaction and the antioxidant activity of the tannin sample was calculated as Trolox equivalents.

### 4.8. Protein Precipitation

Bovine serum albumin (BSA) and chicken egg white lysozyme (LYS) were dissolved in 50 mM MES (2-[*N*-morpholino]ethanesulfonic acid), pH 6.5 (with NaOH) to a concentration of 5 mg/mL. Protein concentration of the desalted alfalfa leaf extract [46] was determined using the Pierce 660 nm Protein Assay Reagent (Thermo Fisher Scientific, Waltham, MA, USA) with BSA as the standard. Small aliquots (<1 mL) of the protein solutions were flash frozen in liquid nitrogen and stored at −80 °C until needed.

A master stock solution of each CT fraction was prepared by weighing out approximately 10 mg of purified CT and dissolving to a final concentration of 12 mg/mL in 50 mM MES, pH 6.5. Further working stock solutions were prepared from the master stock to give 8.00, 6.00, 4.00, 3.00, 2.50, 2.00, 1.50, 1.00, 0.75, 0.625, 0.50, 0.375, 0.25, and 0 mg/mL solutions. For each protein tested, 20 μL of each CT working stock solution (or buffer control for no CT) was pipetted into 1.7 mL microfuge tube containing buffer in duplicate. Previously frozen protein stock solutions were thawed and added to the CT in each tube to give a final reaction volume of 100 μL and a final protein concentration of 1 mg/mL (20 μL for BSA or LYS or ALF). The precipitation reactions were incubated for 30 min then centrifuged for 5 min at 10,000× *g*. Protein present in the supernatant was determined by adding 750 μL Pierce 660 nM protein assay reagent to a supernatant aliquot (50 μL) and measuring absorbance at 660 nm using a Beckman DU800 spectrophotometer (Beckman-Coulter, Brea, CA, USA). Background absorbance due to reaction of the CT with the reagent was recorded. Protein precipitation data were fitted to estimate PP50 defined as the mg of CT for precipitation of 50% of maximal protein precipitated (sometimes referred to as *b* values). After averaging duplicate points, replicate (*n* = 2) experiments of each tannin/protein combination were fit to an inhibitor dose-response curve (log [tannin concentration] versus response with variable slope) model:

logy = A + [(B − A)/(1 + ((X^Hillslope^)/(PP50^Hillslope^)))] (1)

using Prism (GraphPad Software, La Jolla, CA, USA). The fitted curves had R^2^ values of > 0.92 (BSA), > 0.98 (LYS), and > 0.95 (ALF).

## Figures and Tables

**Table 1:** Dry land Texas legumes (*Fabaceae*) used in this study.

Plant	Subfamily	Subtribe	λ_max_ in HCl-Methanol (nm)	Methane Production ^1^ (g/kg DM)
*Desmodium paniculatum*	Papilionoideae	-	543	7.9
*Lespedeza stuevei*	Papilionoideae	-	543	4.9
*Lespedeza cuneata*	Papilionoideae	-	547	15.1
*Mimosa strigillosa*	Mimosoideae	Mimoseae	547	7.6
*Desmanthus illinoensis*	Mimosoideae	Mimoseae	547	24.9
*Neptunia lutea*	Mimosoideae	Mimoseae	547	19.7
*Leucaena retusa*	Mimosoideae	Mimoseae	538	40.7
*Acacia angustissima* STP5	Mimosoideae	Acacieae	508	0.6
*Acacia angustissima* STX	Mimosoideae	Acacieae	505	0.8

^1^ Data from in vitro fermentations, in g methane per kg dry matter (DM) [23].

**Table 2:** Comparison of structural information obtained from thiolysis and ^1^H-^13^C HSQC NMR.

Plant Sample	PC/PD Ratio		*cis*/*trans*			% Galloyl			mDP	
	Thiol	NMR	Thiol	NMR H/C-4	NMR H/C-2	Thiol	NMR H/C-4	NMR H/C-2′,5′	Thiol	NMR
*Desmodium paniculatum*	55/45	52.5/47.5	90/10	87.8/12.2	84.2/15.8	None	None	None	18.6	ND ^1^
*Lespedeza stuevei*	42/58	41.4/58.6	44/56	34.8/65.2	33.9/66.1	3	2.4	1.0	9.3	6.7
*Lespedeza cuneata*	8/92	4.3/95.7	80/20	82.1/17.9	75.4/24.6	None	ND ^2^	5.3	10.6	9.1
*Mimosa strigillosa*	15/85	15.3/84.7	89/11	ND ^3^	ND ^4^	40	50.8	ND ^5^	7.6	6.1
*Desmanthus illinoensis*	3/97	1.8/98.2	98/2	ND ^3^	96.2/3.8	75	76.2	87.5	6.0	5.97
*Neptunia lutea*	12/88	8.2/91.8	93/7	ND ^3^	91.5/8.5	32	34.4	25.6	11.5	8.1
*Leucaena retusa*	99/1	98.6/1.4	98/2	ND ^3^	ND ^4^	29	21.3	34.3	39.2	6.3

ND, not determined. ^1^ Low signal to noise ratio on terminal C-H cross-peak signal. ^2^ Low signal to noise ratio for H/C-4 cross-peak signal. ^3^ Due to galloylation of the CT sample, *cis*/*trans* assignments become ambiguous using H/C-4 cross-peaks. ^4^ Low signal to noise ratio for the *trans* H/C-2 cross-peak signal. ^5^ Integration of peaks indicated > 100 mol % galloylation.

**Table 3:** Interval between clusters of peaks in MALDI spectra ^1^.

Flavan-3-ol	-	(Epi)afzelechin	(Epi)catechin	Epi(gallocatechin)	
5-Deoxy Flavan-3-ol	Guibourtinidol	Fisetinidol	Robinetinidol	-	Gallate Ester
Plant Sample	256.07	272.07	288.06	304.06	152.01
*Desmodium paniculatum*	-	-	++	++	-
*Lespedeza stuevei*	-	-	++	++	+
*Lespedeza cuneata*	-	-	+	++	-
*Desmanthus illinoensis*	-	-	+	++	++
*Neptunia lutea*	-	+	+	++	+
*Leucaena retusa*	-	-	++	-	+
*Acacia angustissima* ^2^	+	++	+	-	+

^1^ CT from *Mimosa strigillosa* was not available when the analysis was performed. ^2^ Both ecotypes of *Acacia* had the same peaks on MALDI-MS.

**Table 4:** Selected MALDI signals and their exact assignments. The polymers comprise the flavan-3-ol subunits (epi)catechin (cat), (epi)gallocatechin (gallocat); the 5-deoxy flavan-3-ol subunits guibourtinidol (gui), fisetinidol (fis) and, in some cases, gallate esters (gallate) ^1^.

Plant Sample	Observed Mass	Formula	Exact Mass	Error (ppm)	Interpretation
*Desmodium paniculatum*	2065.43585	C_105_H_85_O_45_	2065.43630	0.22	cat_4_-gallocat_3_
*Lespedeza stuevei*	1473.31433	C_75_H_61_O_32_	1473.31461	0.19	cat_3_-gallocat_2_
*Lespedeza cuneata*	1825.36676	C_90_H_73_O_42_	1825.35766	4.99	gallocat_6_
*Desmanthus illinoensis*	1369.22063	C_66_H_49_O_33_	1369.21562	3.66	gallocat_3_-gallate_3_
*Neptunia lutea*	3025.55520	C_135_H_125_O_80_	3025.55958	1.45	cat-gallocat_8_-gallate_2_
*Leucaena retusa*	1745.34814	C_89_H_69_O_38_	1745.34670	0.83	cat_5_-gallate_2_
*Acacia angustissima* ^2^	1241.29289	C_67_H_53_O_24_	1241.29269	0.17	cat-gui-fis_2_-gallate

^1^ CT from *Mimosa strigillosa* was not available when the analysis was performed. ^2^ Both ecotypes of *Acacia* had the same peaks on MALDI-MS.

## Supplementary Materials

Table S1. Detailed thiolysis data for the CT used in this study. Table S2. ORAC data for antioxidant capacity for CT used in this study. Table S3. Protein precipitation data for CT used in this study. Tables S4 and S5. Ranges of cross peak NMR signals. Figures S1–S7. NMR spectra for the CT used in this study. Figures S8 and S9. NMR spectra for the *Acacia* CT. Figure S10. Protein precipitation data for the CT used in this study. Figure S11. Protein precipitation versus methane production correlation graphs.
