# Supplementary material for: Relationships between Structures of Condensed Tannins from Texas Legumes and Methane Production During In Vitro Rumen Digestion
Source: Molecules. 2018 Aug 23;23(9):2123. doi: 10.3390/molecules23092123 (PMC6225215; doi:10.3390/molecules23092123)
Supplement: Supplementary file 1 [file molecules-23-02123-s001.pdf]

Supplemental Information

Relationships between Structures of Condensed Tannins from Texas Legumes and Methane Production During *in vitro* Rumen Digestion

Harley Naumann [1], Rebecka Sepela [2], Aira Rezaire [2], Sonia E. Masih [2], Wayne E. Zeller [3], Laurie Reinhardt [3], Jamison T. Robe [3], Michael L. Sullivan[3] and Ann E. Hagerman [2,*]

[1] Division of Plant Sciences, University of Missouri, 110 Waters, Columbia, MO 65211 USA
[2] Department of Chemistry & Biochemistry, Miami University, Oxford, OH 45056 USA; hagermae@miamioh.edu
[3] USDA-ARS, U.S. Dairy Forage Research Center, Madison, WI 53706 USA
* Correspondence: hagermae@miamioh.edu; Tel.: +1-513-529-2827

Supplementary Text

Structure vs. Methanogenesis

Linear correlations between various structural features and methane production were tested for all the plants except Acacia. Limited structural information on Acacia made it impossible to include this unusual CT in the analysis. None of the correlations suggested a strong relationship between any simple structural feature and methanogenesis: chain lengeth ($R^2 = 0.40$),% trans ($R^2 = 0.31$), PC/PD ($R^2 = 0.47$), % galloyl ($R^2 = 0.09$).

NMR Analysis of Purified CTs

Integration of 2D NMR cross-peaks and General Remarks. For each determination, three sets of integration of NMR cross-peak signals were obtained, averaged and a standard deviation was determined. In mDP estimations, we have found that the terminal H/C-4 cross-peaks integrate for, on average, only about 72% of the other cross-peak signals. Thus, to get the estimate of actual volume of the terminal H/C-4 cross-peaks, we take the observed volume, divide it by 2 (since these signals are arising from two C-H signals) and then divide the resulting value by 0.72. The adjustment can also be made by simply taking the observed volume and multiplying by 0.694. This operation then gives you the corrected terminal H/C-4 cross-peak volume. This value is then divided into the value of the extender H/C-4 volume plus the corrected terminal H/C-4 volume. The cis/trans ratios were assigned using both the H/C-4 and H/C-2 cross-peak signals. These estimates require no modification of observed cross-peaks volumes. To calculate % cis isomer, simply take the observed volume of the cis H/C-4 or H/C-2 cross-peak signals and divide them by the combination of the cis plus trans cross-peak signal volumes for H/C-4 or H/C-2, respectively. Estimation of PC/PD ratios were obtained using a combination of integrations of B-ring cross-peak signals from the PC H/C-6′ and PD H/C-2′,6′ subunits. The volume of the integration from the PD subunits is divided by two as it arises from two C-H bond signals. The calculation for %PC then become volumes of the PC H/C-6 divided by the volumes of PC H/C-6 plus one-half the volume of PD H/C-2,6 cross-peaks times 100. The PC/PD ratio can also be estimated by taking the volume of integration of the B-ring cross-peak signals from the PC H/C-2′, 5′ and PD H/C-2′,6′ subunits. In this case no adjustment is need since these cross-peaks arise from 2 C-H signal each. Percent PC would be calculated by taking the volume of the PC H/C-2′, 5′ signal and dividing it by the combined volumes of the PC H/C-2′, 5′ and PD H/C-2′,6′ cross-peak signals time 100. Calculation of the percent galloylation was approached in two ways. Direct integration of the volume of the H/C-4 galloylated cross-peak signal divided by the combination of the cross-peak volumes of the H/C-4 galloylated and H/C-4 non-galloylated signals time 100 provides % galloylation. An additional method includes using the aromatic C-H cross-peak signals. The percent galloylation can be estimated by taking the volume of the galloyl-2″,6′ cross-peaks and dividing by the combination of the volumes of the PC H/C-2′, 5′ and PD H/C-2′,6′ cross-peak signals time 100. Deviation from the above procedures occur on an individual basis and are noted in the specific analysis of each CT.

Ranges of ^{1}H-^{13}C Cross-peak Signals (Tables S4-S5). The purified CT samples from each of the plant sources contain mixtures of millions or even billions of distinct CT chemical entities, with cross-peak (^{1}H and ^{13}C) signals that vary depending on the identity of the flavan-3-ol subunit, the degree of polymerization, the bonding arrangement to and the identity of neighboring flavan-3-ol subunits, and on where the subunit occurs in the CT chain. This gives rise to a set of clustered cross-peaks that appear in a small range of ^{1}H and ^{13}C chemical shifts, indicative of the slightly different electronic environments in which they reside.

2D NMR Analysis of purified CT from *Desmodium paniculatum* (Figure S1). The mDP of this sample could not assigned by 2D NMR due to insufficient cross-peak signal intensity arising the terminal methylene C-H bonds. *Cis*/*trans* ratio determinations for this sample are: For H/C-4 signals

(Panel S1A), 87.8:12.2 (SD ± 0.6); for H/C-2 signals (Panel S1B), 84.2:15.8 (SD ± 0.5). Calculation for the PC/PD (Panel S1C) for the *Desmodium paniculatum* CT is 52.5:47.5 (SD ± 0.4).

2D NMR Analysis of purified CT from *Lespedeza stuevei* (Figure S2). Calculation of the mDP of the *Lespedeza stuevei* CT (Panel S2A): mDP 6.7 (SD ± 0.1). *Cis/trans* ratio determinations for this sample are: For H/C-4 signals (Panel S2B), 34.8:65.2 (SD ± 1.4); for H/C-2 signals (Panel S2C), 33.9:66.1 (SD ± 0.7). Calculation for the PC/PD for the *Lespedeza stuevei* CT is 41.4:58.6 (SD ± 0.5).

2D NMR Analysis of purified CT from *Lespedeza cuneata* (Figure S3). Due to interference of the solvent peak (DMSO) with the upfield terminal H/C-4 cross-peaks, we used only the downfield terminal H/C-4 cross-peaks for the calculation. Thus, the observed volume was not divided by two. Calculation of the mDP of the *Lespedeza cuneata* CT (Panel S3A) : mDP 9.07 (SD ± 0.41). *Cis/trans* ratio determinations for this sample are: For H/C-4 signals (Panel S3B), 82.1:17.9 (SD ± 1.6); for H/C-2 signals (Panel S3C), 75.4:24.6 (SD ± 0.5). Calculation for the PC/PD ratio (Panel S3D) for the *Lespedeza cuneata* CT sample is 4.3:95.7 (SD ± 0.5) and calculation for the % galloylation (Panel S3E) gave 5.3% (SD ± 0.2).

2D NMR Analysis of purified CT from *Mimosa strigillosa* (Figure S4). Calculation of the mDP of the *Mimosa strigillosa* CT (Panel S4A): mDP 6.08 (SD ± 0.03). Calculation of the % galloylation using H/C-4 signals (Panel S4B): 50.8% (SD ± 0.9). Calculation for the PC/PD ratio for the *Mimosa strigillosa* CT (Panel S4C) is 84.7:15.3 (SD ± 0.4). Calculation of the % galloylation using PD H/C-2′,6′, PC H/C-2′,5′ and galloyl-2″,6″ signals (Panel S4D): 107% (SD ± 5).

2D NMR Analysis of purified CT from *Desmanthus illinoensis* (Figure S5). Calculation of the mDP of the *Desmanthus illinoensis* CT (Panel S5A): mDP 5.97 (SD ± 0.3). Calculation of the % galloylation using H/C-4 signals (Panel S5B): 76.2% (SD ± 0.8). *Cis/trans* ratio determination for this sample for H/C-2 signals (Panel S5C), 96.2:3.8 (SD ± 0.1). Calculation for the PC/PD ratio for the *Desmanthus illinoensis* CT (Panel S5D) is 1.8:98.2 (SD ± 0.1). Calculation of the % galloylation using PD H/C-2′,6′, PC H/C-2′,5′ and galloyl-2″,6″ signals (Panel S5E): 87.5% (SD ± 1.6).

2D NMR Analysis of purified CT from *Neptunia lutea* (Figure S6). Calculation of the mDP of the *Neptunia lutea* CT (Panel S6A): mDP 8.1 (SD ± 0.2). Calculation of the % galloylation using H/C-4 signals (Panel S6B): 34.4% (SD ± 1.6). *Cis/trans* ratio determination for this sample for H/C-2 signals (Panel S6C), 91.5:8.5 (SD ± 0.1). Calculation for the PC/PD ratio for the *Neptunia lutea* CT (Panel S5D) is 1.8:91.8 (SD ± 0.8). Calculation of the % galloylation using PD H/C-2′,6′, PC H/C-2′,5′ and galloyl-2″,6″ signals (Panel S5E): 25.6% (SD ± 1.1).

2D NMR Analysis of purified CT from *Leucaena retusa* (Figure S7). Calculation of the mDP of the *Leucaena retusa* CT (Panel S7A): mDP 6.25 (SD ± 0.3). Calculation of the % galloylation using H/C-4 signals (Panel S7B): 21.3% (SD ± 1.0). Calculation for the PC/PD ratio for the *Leucaena retusa* CT (Panel S7C) is 98.6:1.4 (SD ± 0.2). Calculation of the % galloylation using PD H/C-2′,6′, PC H/C-2′,5′ and galloyl-2″,6″ signals (Panel S7D): 34.3% (SD ± 1.0).

Supplementary Tables

Table S1. Detailed thiolysis data. Data is normalized per average chain of the polymer, so for each plant terminal units sum to 1.0 and extender plus terminal units sum to the mDP [1,2].

		D.pan	***L.stu***	***L.cun***	***Mim***	***D.ill***	***Nep.***	***Leu.***
mDP		18.6	9.3	10.6	7.6	5.0	11.5	39.2
Extenders per chain	GC	0.2	1.8	1.0	0.2	0.1	0.2	0.0
	EGC	8.1	2.7	7.7	3.2	1.3	6.2	0.5
	Cat	0.6	2.2	0.4	0.3	0.0	0.0	0.0
	EC	8.7	1.3	0.4	0.5	0.1	0.8	26.4
	GCg	0.0	0.3	0.0	0.0	0.0	0.0	0.0
	EGCg	0.0	0.0	0.0	2.2	3.5	2.8	0.0
	Cg	0.0	0.0	0.0	0.1	0.0	0.2	0.6
	ECg	0.0	0.0	0.0	0.2	0.0	0.3	10.7
Terminal per chain	GC	0.1	0.5	0.6	0.2	0.0	0.3	0.0
	EGC	0.0	0.1	0.3	0.0	0.0	0.2	0.0
	Cat	0.9	0.4	0.1	0.1	0.0	0.1	0.0
	EC	0.0	0.0	0.0	0.0	0.0	0.0	1.0
	EGCg	0.0	0.0	0.0	0.7	1.0	0.4	0.0

[1] Plant names are *Desmodium paniculatum (D. pan), Lespedeza stuevei (L.stu), Lespedeza cuneate (L.cun), Mimosa strigillosa (Mim.), Desmanthus illinoensis (D.ill), Neptunia lutea (Nep.), Leucaena retusa (Leu.)*.

[2] Compound names are gallocatechin (GC), epigallocatechin (EGC), catechin (Cat), epicatechin (EC), gallocatechin gallate (GCg), epigallocatechin gallate (EGCg), catechin gallate (Cg), epicatechin gallate (ECg).

Table S2. Antioxidant potential and levels of CT for the plants in this study [1].

Plant	Trolox equivalents g/g CT	Extractible CT g/100 g DM[2]
Desmodium paniculatum	1.47	10.3
Lespedeza stuevei	1.16	9.9
Lespedeza cuneata	0.73	4.7
Mimosa strigillosa	0.50	9.9
Desmanthus illionensis	0.57	5.1
Neptuia lutea	0.32	7.0
Leucaena retusa	0.49	3.4
Acacia angustissima STP5	0.73	4.9
Acacia angustissima STX	0.89	4.9

[1] CT data from Naumann et al. 2013b. [2] DM, dry matter.

Table S3. PP_{50} Values for condensed tannin from plants for precipitation with proteins BSA, LYS, and ALF.

**Plant**	**PP_{50} (mg tannin / mg protein)[1]**		
	**BSA**	**LYS**	**ALF**
Desmodium paniculatum	0.86 ± 0.08	0.40 ± 0.03	0.32 ± 0.04
Lespedeza stuvei	0.72 ± 0.09	0.37 ± 0.01	0.32 ± 0.04
Lespedeza cuneata	0.77 ± 0.07	0.33 ± 0.01	0.34 ± 0.02
Mimosa strigillosa	0.49 ± 0.01	0.25 ± 0.01	0.33 ± 0.02
Desmanthus illionensis	0.41 ± 0.01	0.29 ± 0.01	0.25 ± 0.04
Leucaena retusa	1.72± 0.06	0.40 ± 0.02	0.48± 0.06
Acacia angustissima	0.72 ± 0.06	0.39 ± 0.02	0.36 ± 0.05

[1]*relative error from least squares nonlinear regression fit of replicate experiments*

Table S4. Range of ^{1}H and ^{13}C chemical shifts for H/C-4 and H/C-2 used in structural determination of cis/trans ratio for series of perennial legumes.

Plant Sample	Isomer or galloylated	H/C-4[1] ^{1}H	H/C-4[1] ^{13}C	Isomer	H/C-2[2] ^{1}H	H/C-2[2] ^{13}C
Desmodium paniculatum	*cis*	4.17 – 4.79	36.0 – 37.5	*cis*	4.47 – 5.78	75.8 – 81.0
	trans	4.58 – 4.19	37.5 – 39.8	*trans*	4.20 – 4.54	80.6 – 83.1
Lespedeza stuevei	*cis*	4.26 – 4.78	35.7 – 37.2	*cis*	4.51 – 5.30	74.2 – 77.0
	trans	4.12 – 4.56	37.2 – 39.1	*trans*	3.93 – 4.86	79.8 – 85.0
Lespedeza cuneata	*cis*	4.13 – 4.79	35.0 – 37.5	*cis*	4.49 – 5.30	72.1 – 79.0
	trans	4.15 – 4.57	37.5 – 38.5	*trans*	4.05 – 4.69	80.6 – 84.0
Mimosa strigillosa	galloylated	4.26 – 4.87[3]	35.2 – 37.4[3]		ND[4]	ND[4]
		4.31 – 5.00[3]	32.7 – 34.8[3]			
Desmanthus illinoensis	galloylated	4.51 – 4.86[3]	35.6 – 37.2[3]	*cis*	4.75 – 5.40	74.0 – 7.50
		4.20 – 5.00[3]	32.5 – 35.6[3]	*trans*	4.75 – 4.84	78.2 – 79.0
Neptunia lutea	galloylated	4.11 – 4.89[3]	34.5 – 38.1[3]	*cis*	4.38 – 5.30	73.9 – 79.9
		4.30 – 4.45[3]	31.0 – 34.5[3]	*trans*	4.20 – 4.85	85.2 – 83.5
Leucaena retusa	galloylated	4.11 – 4.89[3]	35.1 – 37.7[3]		ND[4]	ND[4]
		4.31 – 4.89[3]	32.7 – 34.7[3]			

ND, not determined
[1]Utilized to determine mDP and *cis*/*trans* ratio or percent galloylation.
[2]Utilized to determine *cis*/*trans* ratio.
[3] Not used for *cis*/*trans* ratio as galloylation of the CT sample makes *cis*/*trans* assignments ambiguous using H/C-4 cross-peaks.
[4] Not used for integration due to low signal to noise ratio for the *trans* H/C-2 cross-peak signal.

Table S5. Range of ^{1}H and ^{13}C chemical shifts for H/C-2′,6′and H/C-6′used in structural determination of PC/PD ratio and percent galloylation for series of perennial legumes.

Plant Sample	PC H/C-6′ [1]		PD H/C-2′,6′ [1,2]		H/C-2′,5′ [2]		Galloyl-2′,6′ [2]	
	^{1}H	^{13}C	^{1}H	^{13}C	^{1}H	^{13}C	^{1}H	^{13}C
Desmodium paniculatum	6.45 – 6.92	116.6 – 119.0	6.15 – 6.56	103.0 – 104.8	none	none	none	none
Lespedeza stuevei	5.90 – 6.88	117.0 – 120.4	6.11 – 6.58	104.7 – 108.8	6.48 – 7.03	113.6 – 117.0	6.61 – 6.66	109.1 – 111.0
Lespedeza cuneata	6.52 – 6.75	116.8 – 118.0	5.87 – 6.82	104.0 – 108.0	6.55 – 7.06	114.3 – 118.0	6.66 – 7.18	108.5 – 110.1
Mimosa strigillosa	6.59 – 7.02	114.9 – 116.8	6.01 – 6.82	108.0 – 104.8	ND[3]	ND[3]	6.53 – 7.38	115.0 – 110.5
Desmanthus illinoensis	6.64 – 6.76	117.0 – 118.0	6.64 – 5.60	108.4 – 102.4	6.61 – 6.90	115.0 – 116.3	6.48 – 7.19	107.5 – 110.6
Neptunia lutea	6.52 – 6.85	116.5 – 118.0	5.92 – 6.64	104.0 – 108.5	6.46 – 7.08	113.5 – 116.0	6.52 – 7.11	108.4 – 110.1
Leucaena retusa	6.58 – 7.06	117.6 – 120.0	6.38 – 6.48	105.8 – 106.6	6.11 – 7.23	111.8 – 116.4	6.50 – 7.16	106.9 – 110.2

ND, – not determined
[1] Utilized to determined PC/PD ratio.
[2] Utilized to determine percent galloylation.
[3]Integration of peaks indicated >100 mol % galloylation.

Supplementary Figures

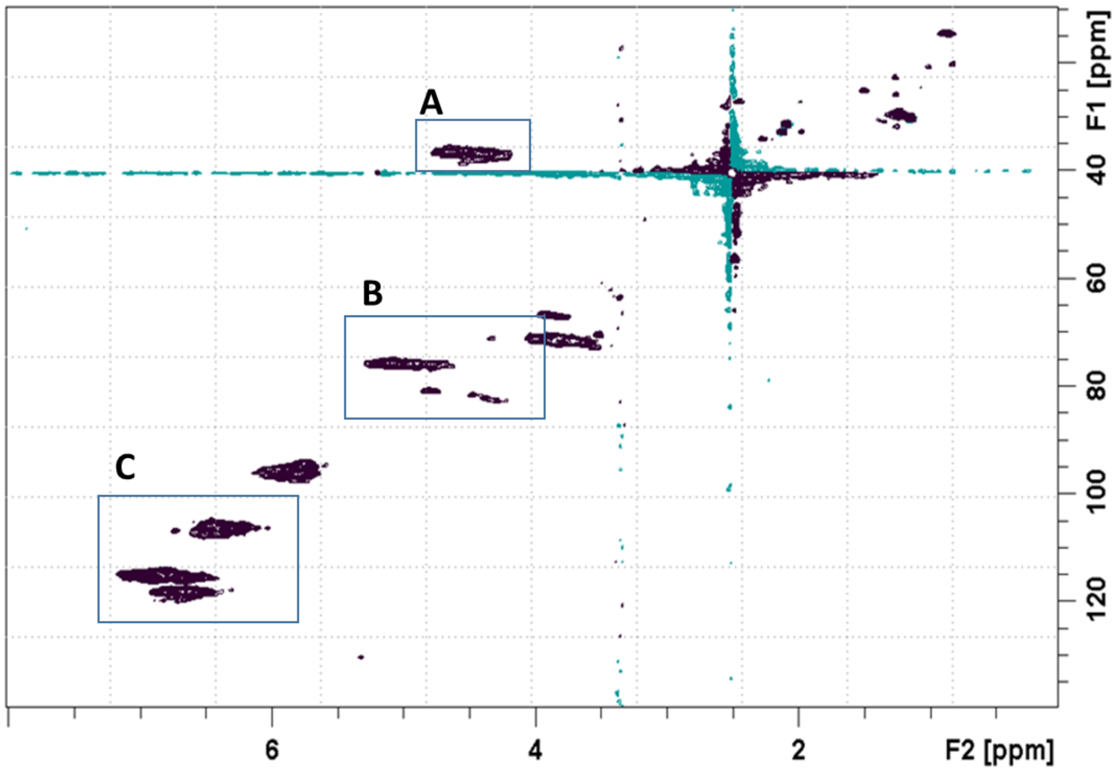

Figure S1. The ^{1}H-^{13}C HSQC NMR spectrum of purified CT from *Desmodium paniculatum*.

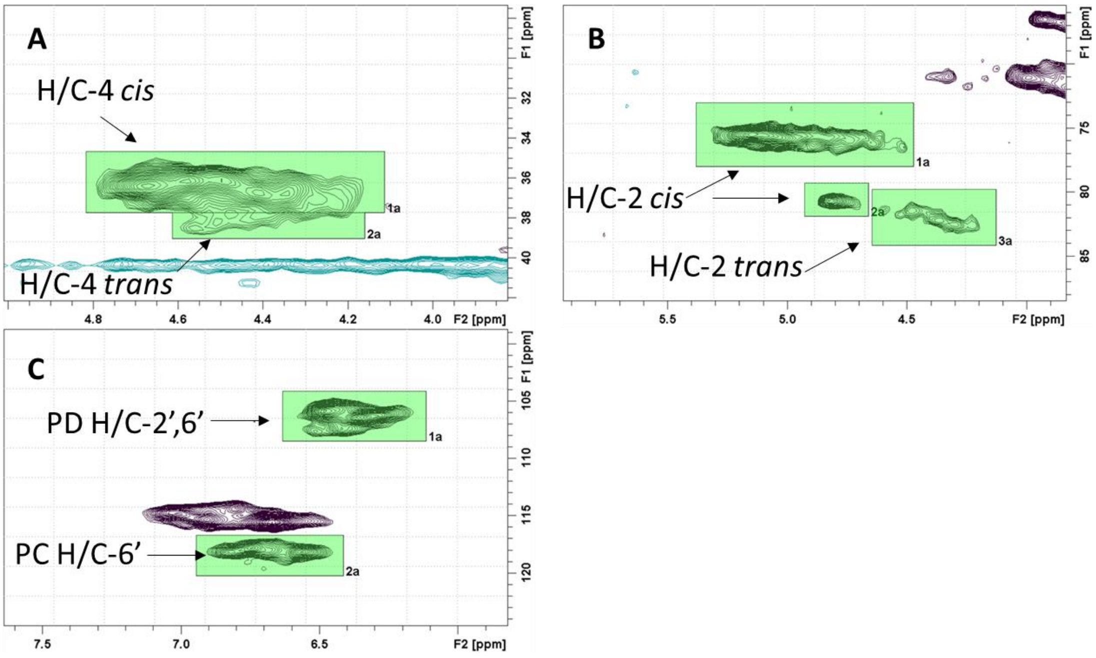

Figure S1A-C. Expanded sections of the ^{1}H-^{13}C HSQC NMR spectrum of purified CT from *Desmodium paniculatum* showing integrated cross-peak signals used for compositional and structural analysis.

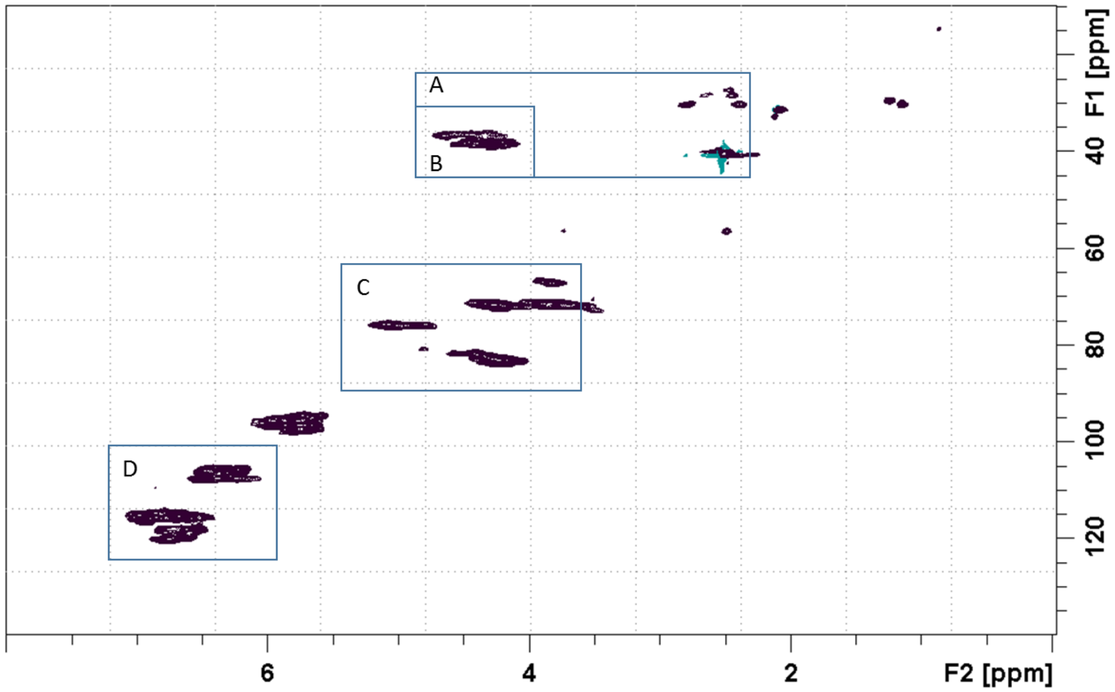

Figure S2. The ^{1}H-^{13}C HSQC NMR spectrum of purified CT from *Lespedeza stuevei.*

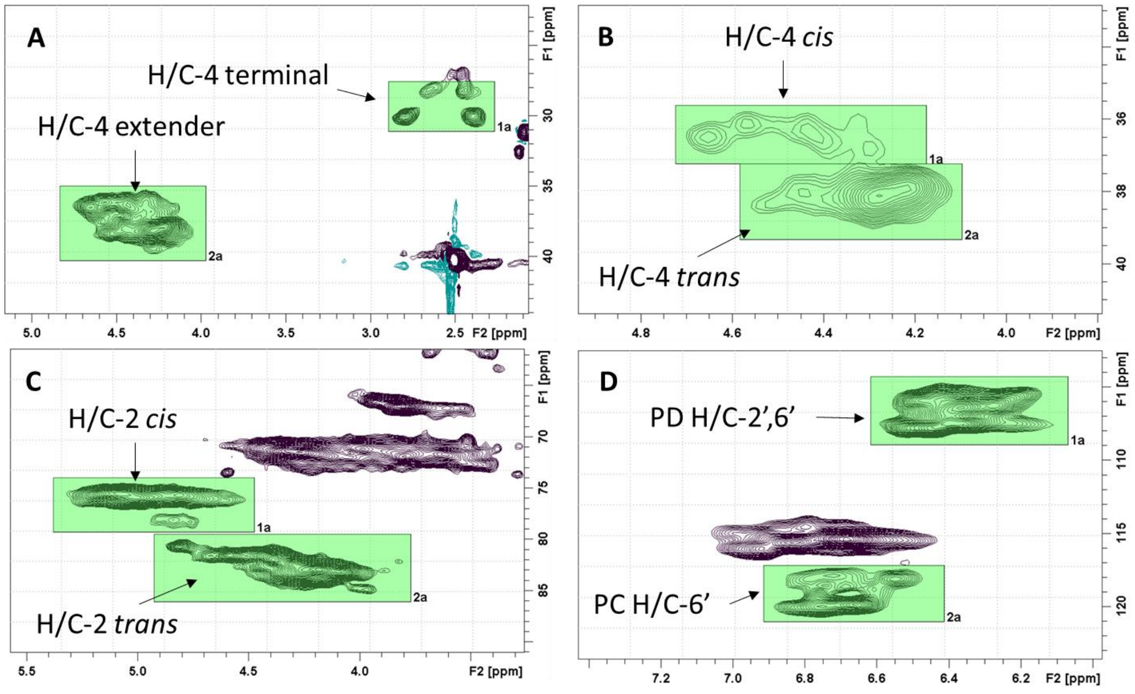

Figure S2A-D. Expanded sections of the ^{1}H-^{13}C HSQC NMR spectrum of purified CT from *Lespedeza stuevei* showing integrated cross-peak signals used for compositional and structural analysis.

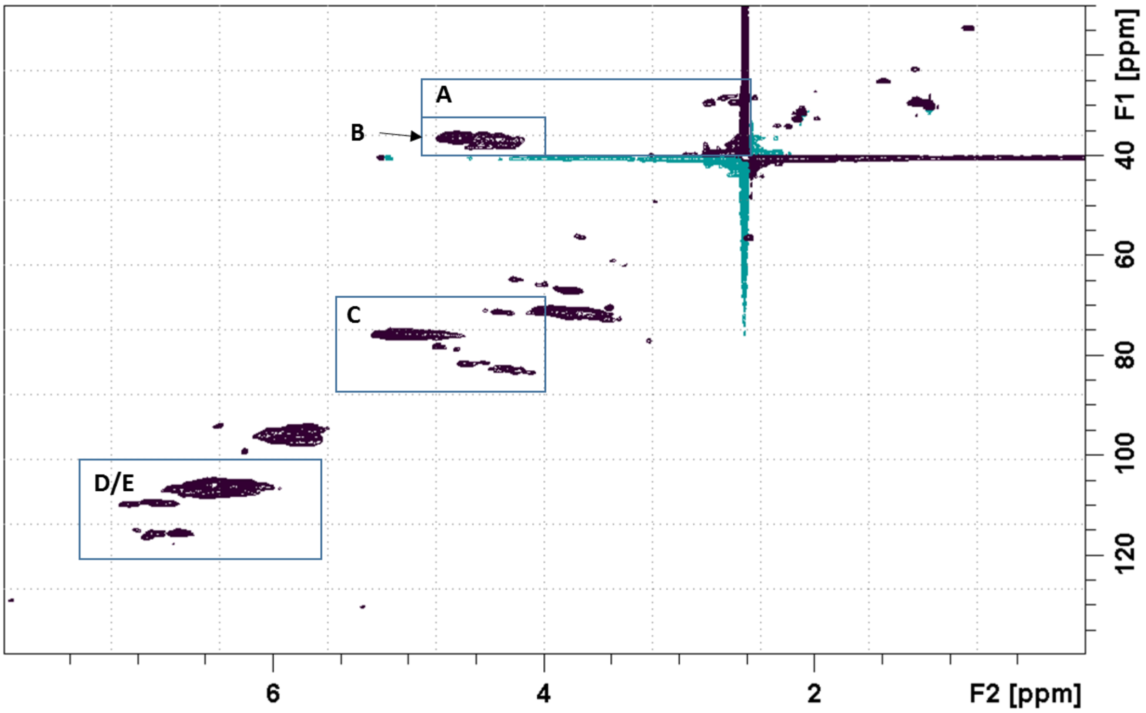

Figure S3. The ^{1}H-^{13}C HSQC NMR spectrum of purified CT from *Lespedeza cuneata.*

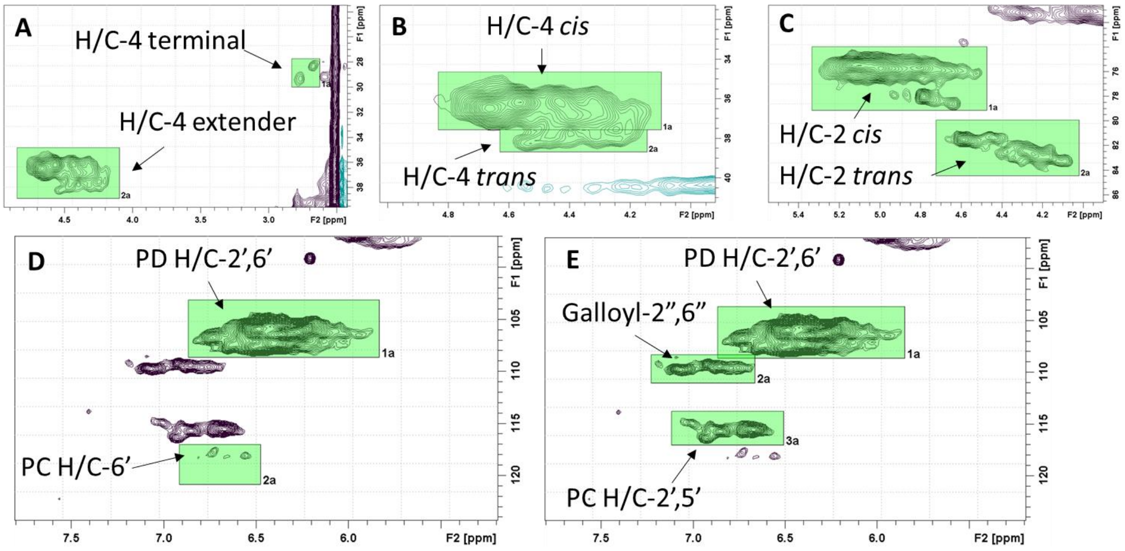

Figure S3A-E. Expanded sections of the ^{1}H-^{13}C HSQC NMR spectrum of purified CT from *Lespedeza cuneata* showing integrated cross-peak signals used for compositional and structural analysis.

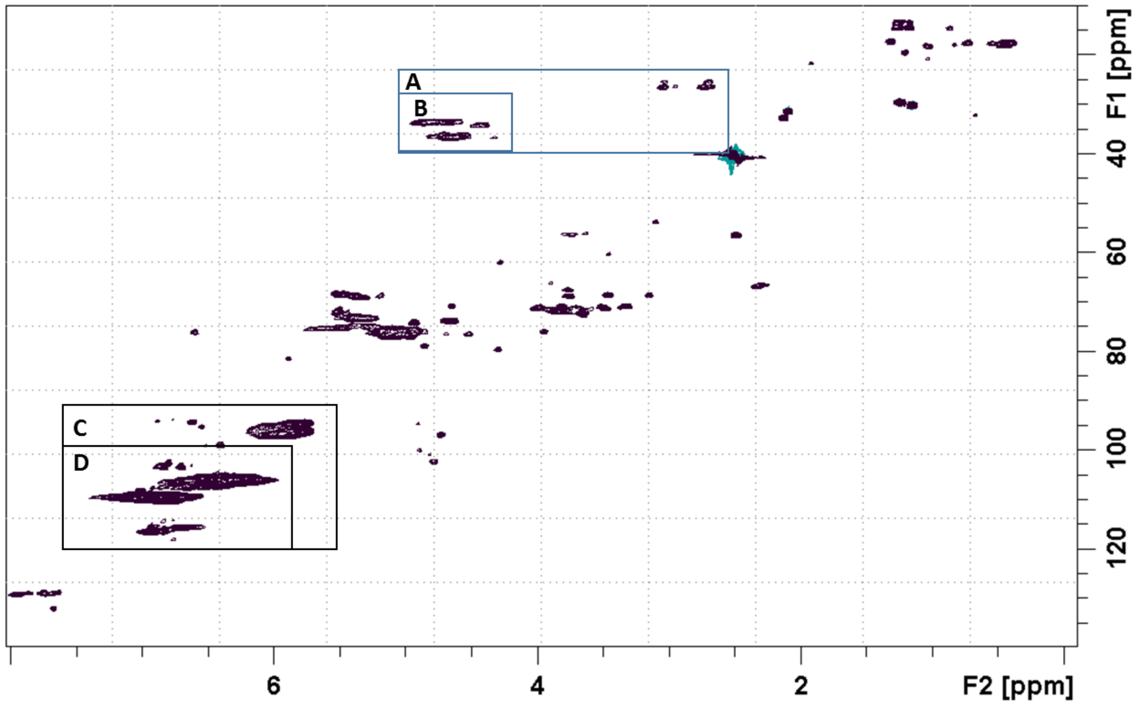

Figure S4. The ^{1}H-^{13}C HSQC NMR spectrum of purified CT from *Mimosa strigillosa.*

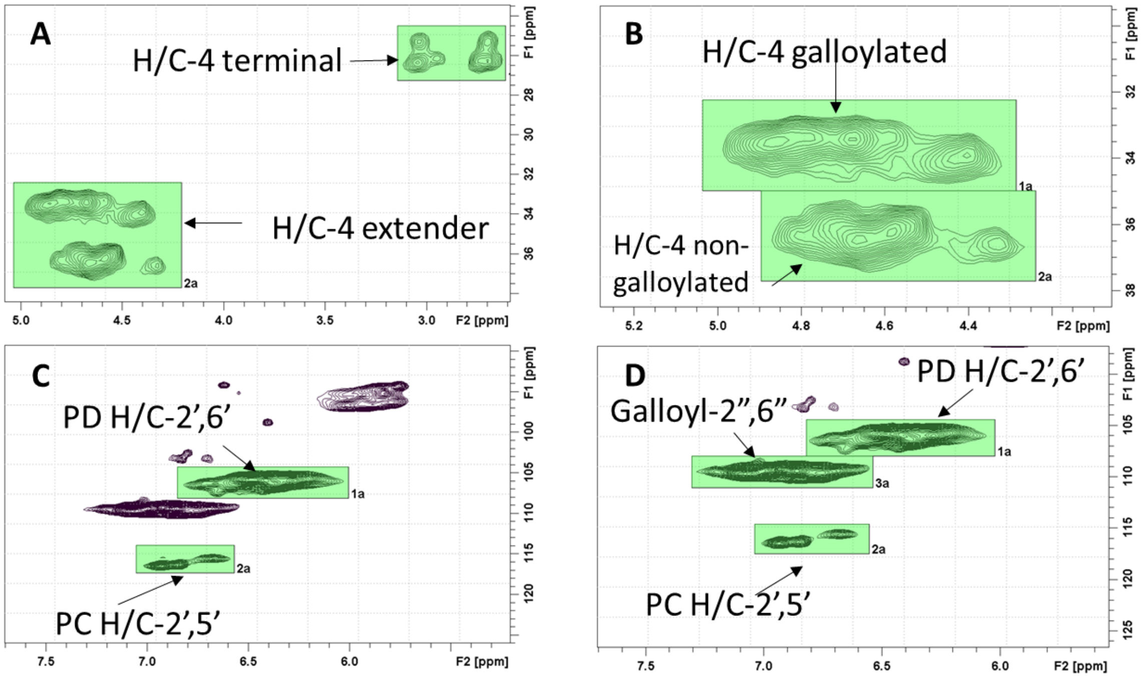

Figure S4A-D. Expanded sections of the ^{1}H-^{13}C HSQC NMR spectrum of purified CT from *Mimosa strigillosa* showing integrated cross-peak signals used for compositional and structural analysis.

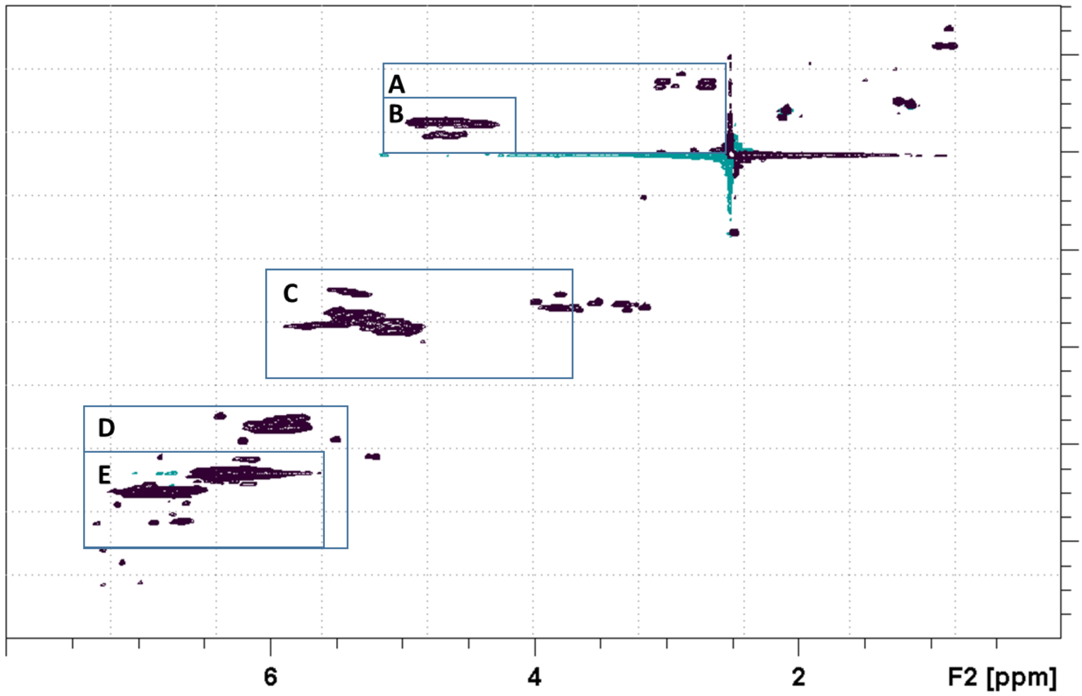

Figure S5. The ^{1}H-^{13}C HSQC NMR spectrum of purified CT from *Desmanthus illinoensis*.

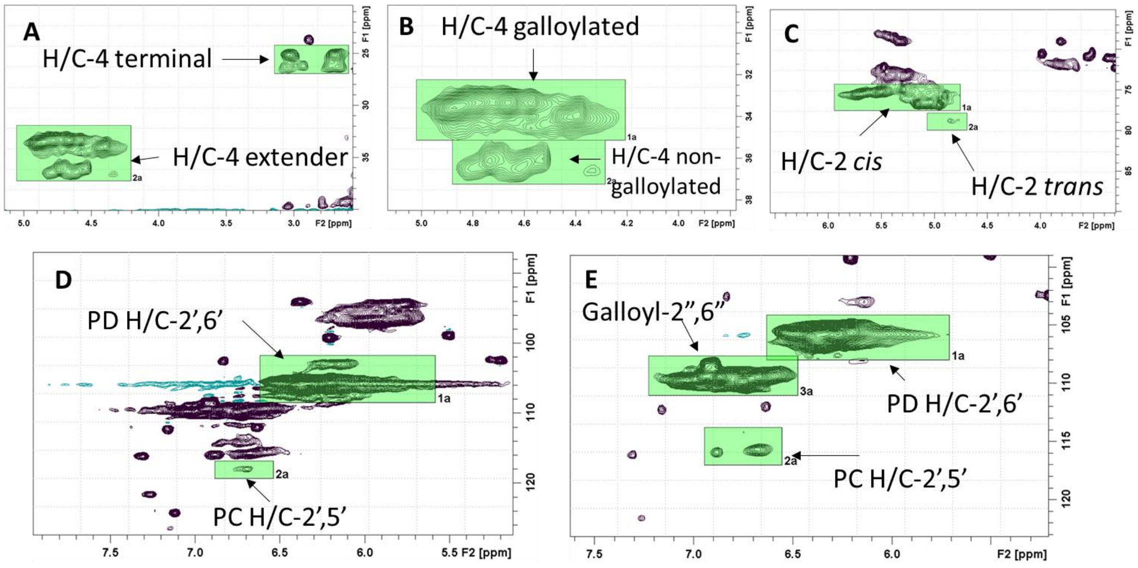

Figure S5A-E. Expanded sections of the ^{1}H-^{13}C HSQC NMR spectrum of purified CT from *Desmanthus illinoensis* showing integrated cross-peak signals used for compositional and structural analysis.

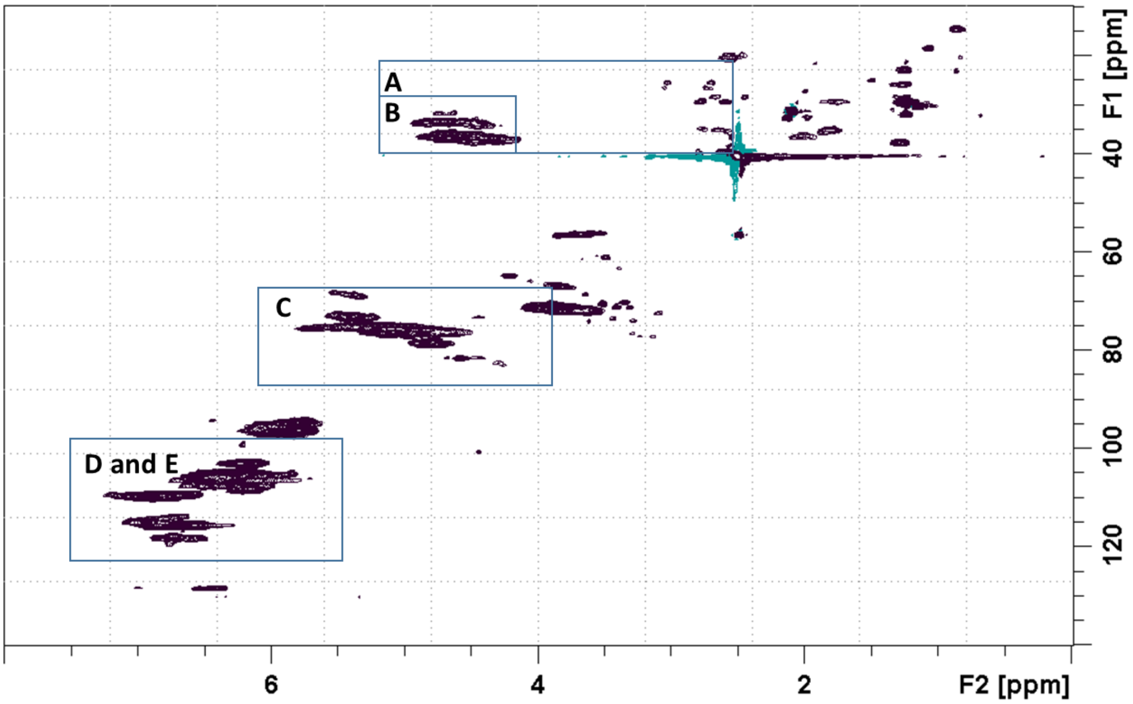

Figure S6. The ^{1}H-^{13}C HSQC NMR spectrum of purified CT from *Neptunia lutea*.

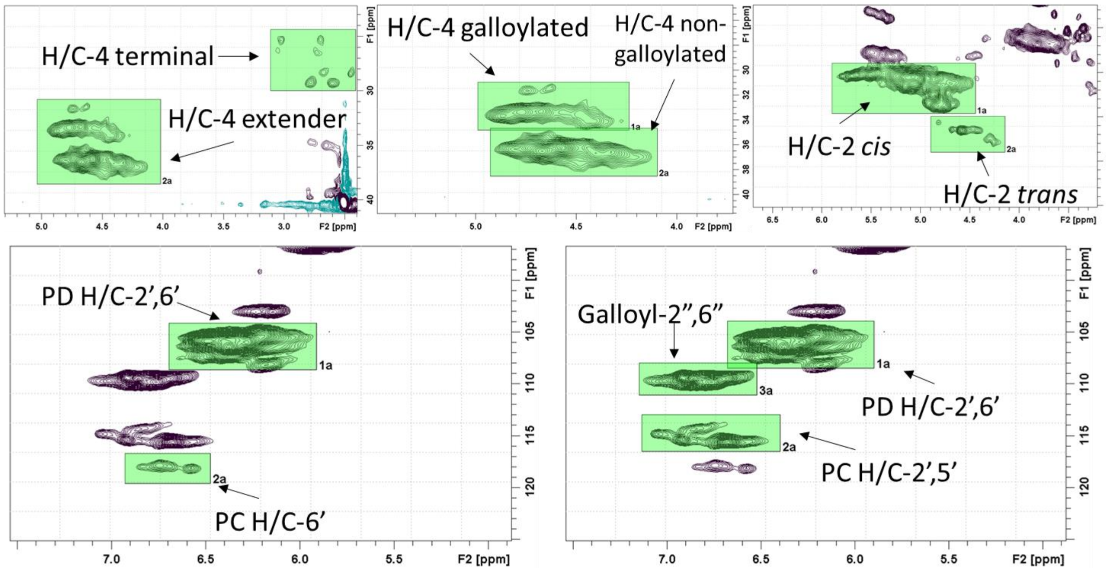

Figure S6A-E. Expanded sections of the ^{1}H-^{13}C HSQC NMR spectrum of purified CT from *Neptunia lutea* showing integrated cross-peak signals used for compositional and structural analysis.

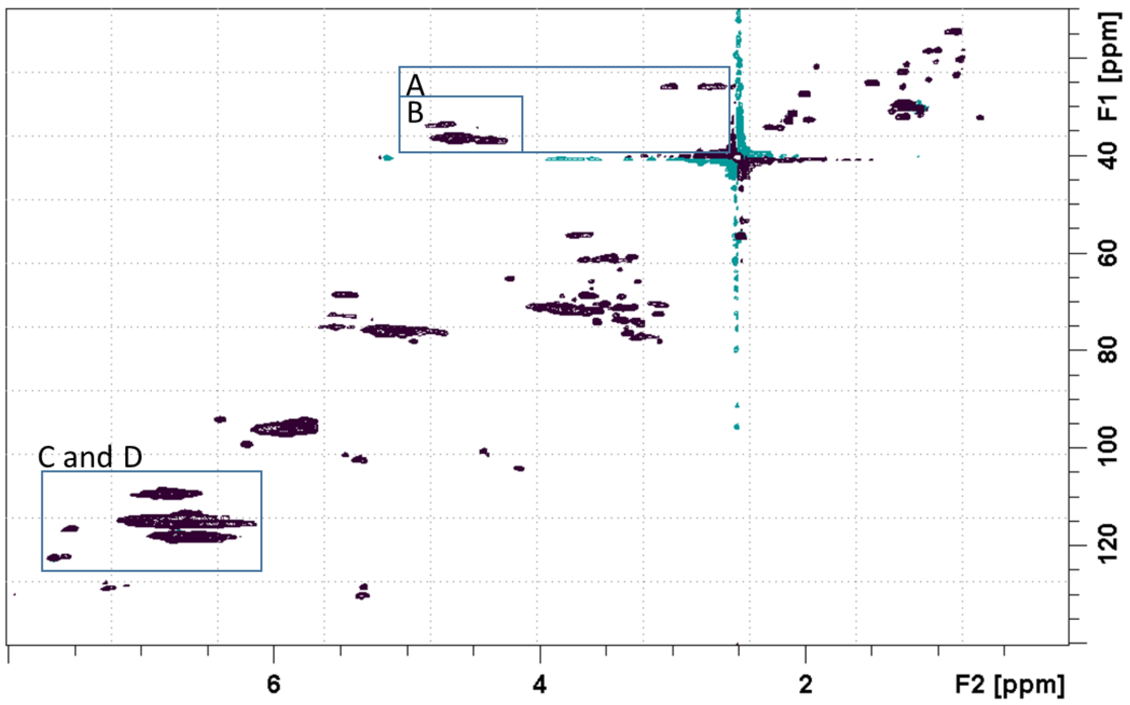

Figure S7. The ^{1}H-^{13}C HSQC NMR spectrum of purified CT from *Leucaena retusa*.

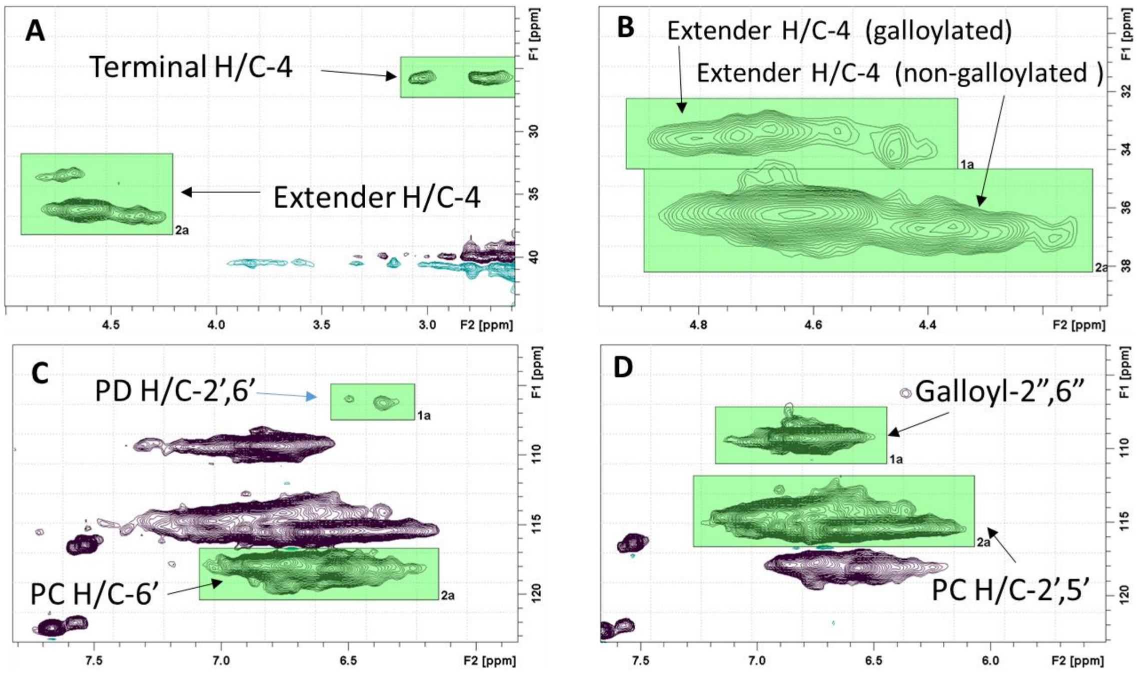

Figure S7A-D. Expanded sections of the ^{1}H-^{13}C HSQC NMR spectrum of purified CT from *Leucaena retusa* showing integrated cross-peak signals used for compositional and structural analysis.

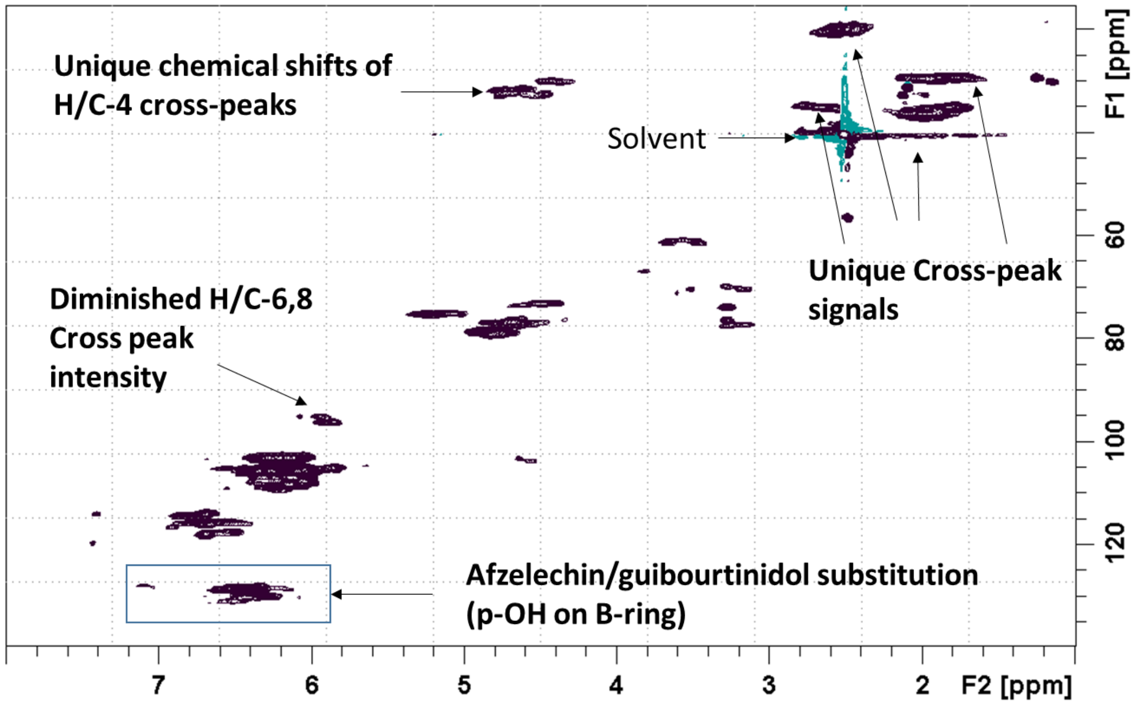

Figure S8. ^{1}H-^{13}C-HSQC NMR spectrum of purified condensed tannins isolated from *Acacia angustissima* (Prairie acacia, South Texas ecotype). Atypical cross-peaks signals are labeled.

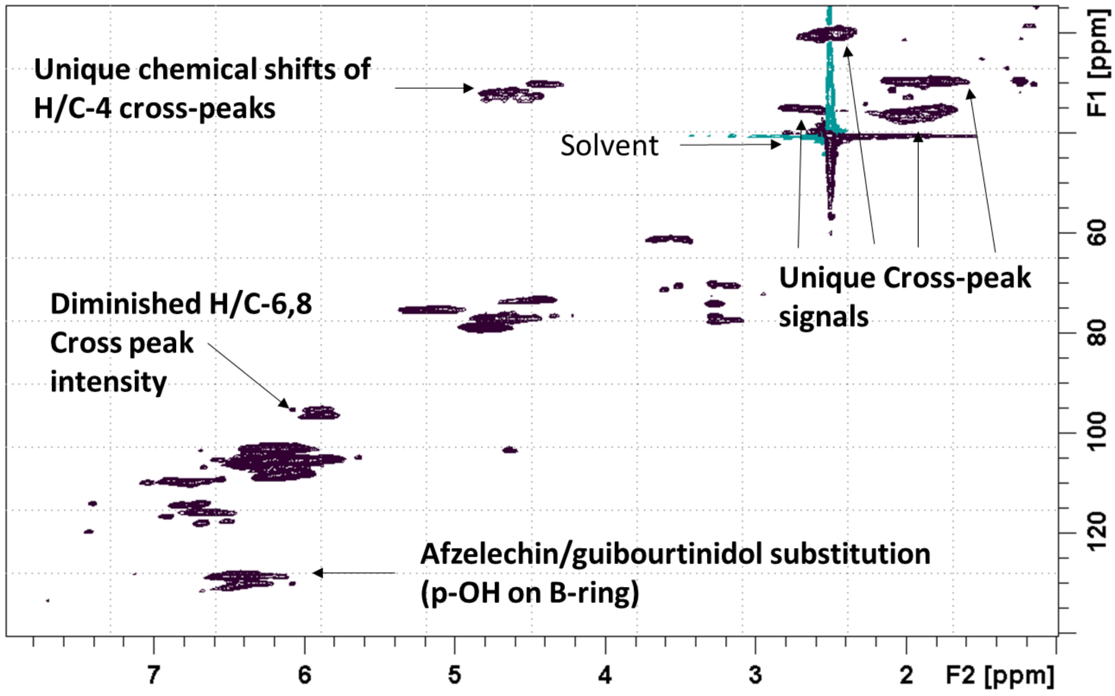

Figure S9. ^{1}H-^{13}C-HSQC NMR spectrum of purified condensed tannins isolated from *Acacia angustissima* (Prairie acacia, Cross Timbers ecotype). Atypical cross-peaks signals are labeled.

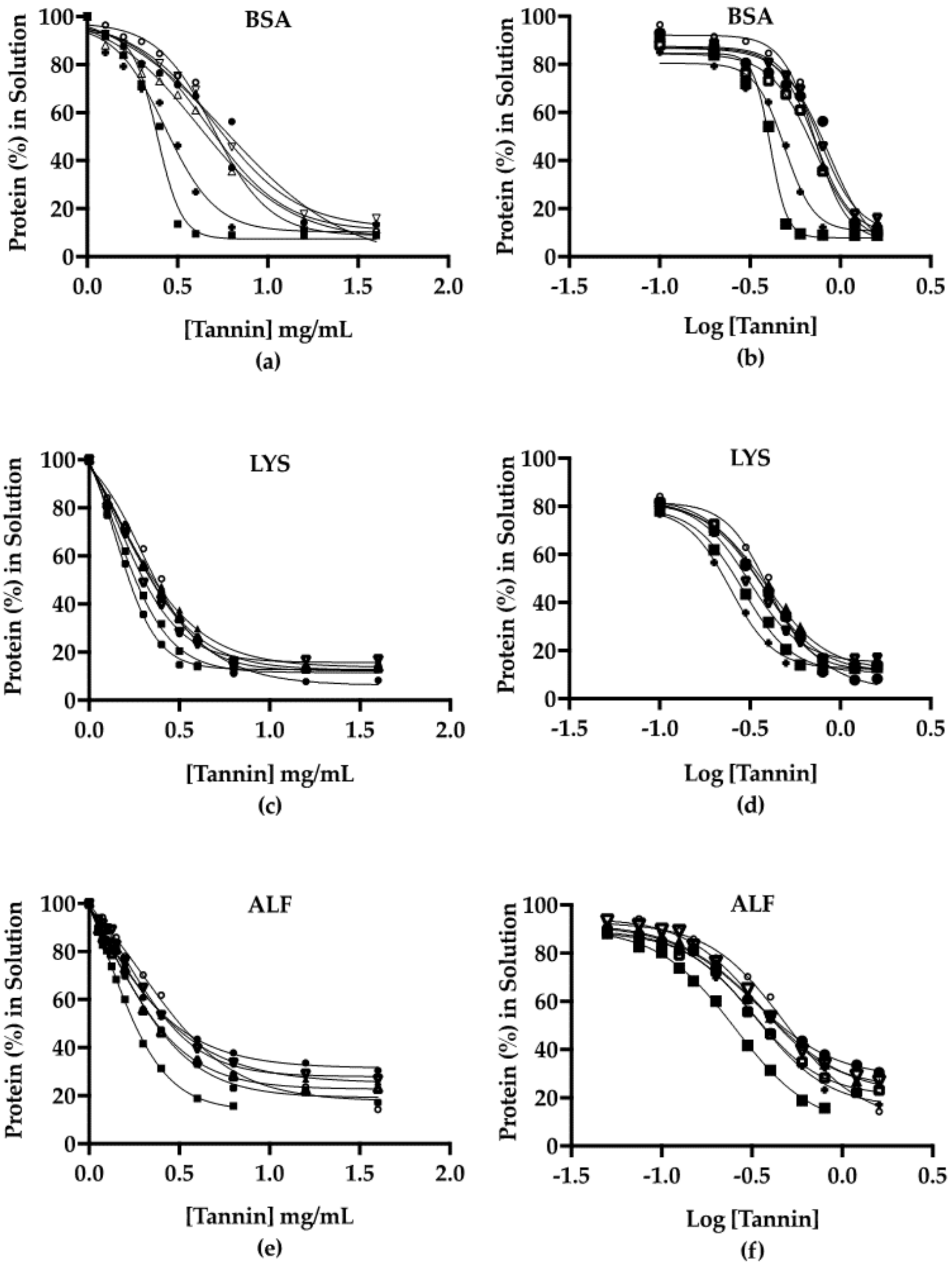

Figure S10. Precipitation. BSA (**a**), LYS (**c**), and ALF (**e**) protein precipitation behavior in the presence of increasing CT (mg/mL) concentrations. CTs were obtained from various forages: *Desmodium paniculatum* (●), *Lespedeza stuevei* (□), *Lespedeza cuneata* (▽), *Mimosa strigillosa* (+), *Desmanthus illionensis* (■),*Leucaena retusa* (○), and *Acacia angustissima* (▲). Precipitation studies were performed using MES buffer, pH 6.5. Data were fit to a log (inhibitor) versus response model and resulting curves are shown in (**b**), (**d**), and (**f**) while PP50 values are listed in **Table S**3.

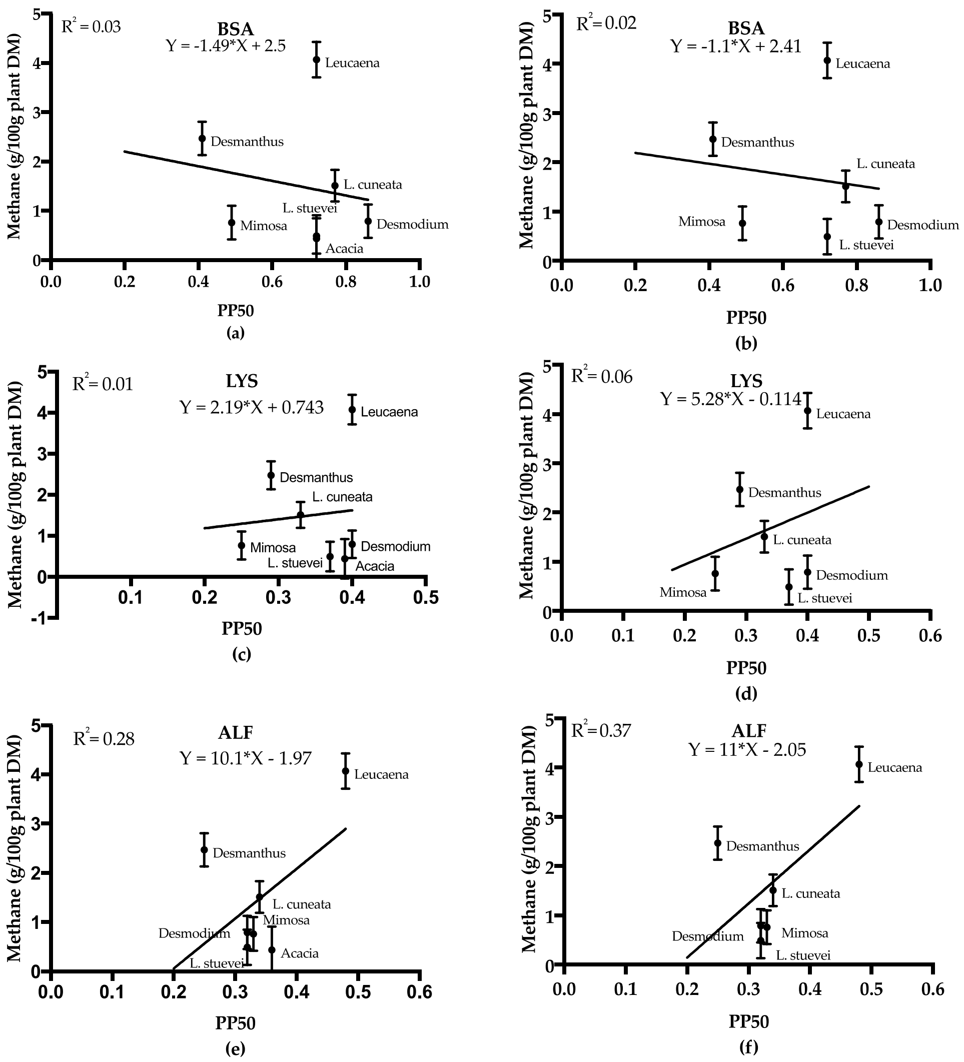

Figure S11. PP50 values for the forages versus methane production [23]. PP50 values were determined using proteins BSA (**a, b**), LYS (**c, d**), and ALF (**e, f**). Correlation coefficients shown in each panel confirm that there is no simple relationship between precipitation and methane production whether the data for *Acacia* were included (**a**, **c**, **e**) or not (**b**, **d**, **f**).
